# HDACs and Their Inhibitors on Post-Translational Modifications: The Regulation of Cardiovascular Disease

**DOI:** 10.3390/cells14141116

**Published:** 2025-07-20

**Authors:** Siyi Yang, Yidong Sun, Wei Yu

**Affiliations:** 1 Institute of Biochemistry, College of Life Sciences and Medicine, Zhejiang Sci-Tech University, Xiasha High-Tech Zone No.2 Road, Hangzhou 310018, China; 2023220902051@mails.zstu.edu.cn (S.Y.); 2023332864030@mails.zstu.edu.cn (Y.S.); 2 Zhejiang Provincial Key Laboratory of Silkworm Bioreactor and Biomedicine, Hangzhou 310018, China

**Keywords:** cardiovascular disease (CVD), post-translational modification (PTM), HDAC, HDACi

## Abstract

Cardiovascular diseases (CVD), such as myocardial hypertrophy, heart failure, atherosclerosis, and myocardial ischemia/reperfusion (I/R) injury, are among the major threats to human health worldwide. Post-translational modifications alter the function of proteins through dynamic chemical modification after synthesis. This mechanism not only plays an important role in maintaining homeostasis and plays a crucial role in maintaining normal cardiovascular function, but is also closely related to the pathological state of various diseases. Histone deacetylases (HDACs) play an important role in the epigenetic regulation of gene expression, and play important roles in post-translational modification by catalyzing the deacetylation of key lysine residues in nucleosomal histones, which are closely associated with the occurrence and development of cardiovascular diseases. Recent studies indicate that HDAC inhibitors (HDACis) may represent a new class of drugs for the treatment of cardiovascular diseases by influencing post-translational modifications. In this review, we systematically summarize the mechanism of action of HDACs and HDACis in post-translational modifications related to common cardiovascular diseases, providing new ideas for the treatment of CVD, and explore possible future research directions on the relationship between HDAC and HDACi in post-translational modifications and cardiovascular diseases.

## 1. Introduction

### 1.1. Cardiovascular Disease

According to the World Health Organization, cardiovascular diseases remain the leading cause of death and impose a tremendous economic burden on society. Studies predict that there will be a 90.0% increase in cardiovascular prevalence and 73.4% increase in the crude death rate between 2025 and 2050, with the number of cardiovascular deaths projected to reach 20.5 million in 2025. Ischemic heart disease will remain the leading cause of cardiovascular death, while high systolic blood pressure will be the major vascular risk factor for death [1]. In addition, as the global population ages [2], the United Nations predicts that by 2050, nearly one in six people will be over 65, while cardiovascular disease places a massive burden on elderly patients [3] and significantly affects their quality of life [4]. Cardiovascular disease involves the heart and blood vessels, including diseases such as atherosclerosis, myocardial remodeling, hypertension, and myocardial ischemia/reperfusion (I/R) injury. Several factors can influence the development and progression of cardiovascular disease, including age, gender, obesity, smoking, alcohol consumption, hypertension, and various psychosocial factors, among others. In recent years, epigenetic modifications have been shown to be involved in the pathophysiological processes of cardiovascular disease [5], including various post-translational modifications. Therefore, studying the relationship between cardiovascular diseases and post-translational modifications of proteins may yield valuable novel insights and therapeutic targets.

### 1.2. Post-Translational Modifications of Proteins

Post-translational modifications (PTMs) are covalent modifications of amino acid side chains in translated proteins, which are catalyzed by various enzymes after the completion of the translation process or during hydrolytic processing and folding [6]. PTM is also essential for epigenetic expression mechanisms, which are related to replication, transcription, organism development, and cell differentiation [7]. PTM enriches the proteome by covalently adding functional groups to one or more amino acid residues of a protein, thereby altering its function and orientation [8,9]. PTM plays a key role in several physiological and cellular processes, including cell differentiation [10], protein degradation [11], signaling [12], the regulation of gene expression [13], and protein–protein interactions [14,15]. Under physiological and pathological conditions, it can expand the functional diversity of proteins by regulating protein folding, activity, stability, localization, signal transduction, and binding [16]. The primary forms of PTMs include ubiquitination, phosphorylation, acetylation, methylation, and glycosylation [8], which have been shown to affect various metabolic pathways and are implicated in the development and progression of various diseases [17]. Recently, novel PTMs, such as succinylation and lactylation, have been discovered. As histones were the first discovered substrates to be modified by acetylation [18], the enzymes responsible for adding and removing acetyl groups are commonly referred to as histone acetyltransferases (HATs) and histone deacetylases (HDACs), respectively. These broad classes of modifying enzymes regulate many different protein modification types, making them attractive targets for drug development.

### 1.3. Histone Deacetylases (HDACs)

**Figure 1:**
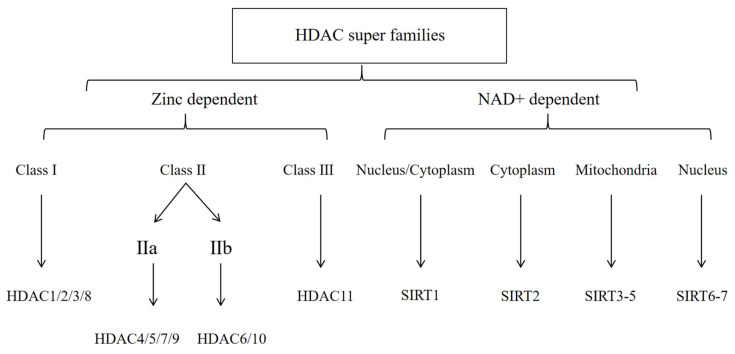
18 mammalian histone deacetylases (HDACs). It was subdivided into four different categories based on phylogenetic analysis, enzyme activity, and structural domain structure.

As the structural units of chromatin, nucleosomes are composed of DNA and histones, which are important for DNA packaging in eukaryotic cells [19]. The nucleosome is composed of an octamer consisting of one H3-H4 tetramer and two H2A-H2B dimers, around which DNA is wrapped [19]. HAT-catalyzed lysine acetylation of histone tails protruding from nucleosomes leads to the relaxation of chromatin structure, creating a conducive environment for transcriptional activation. HDACs exert their catalytic role by removing acetyl groups from lysine residues in histone tails. Thus, when HDACs are more active, histones bind to DNA more tightly, making it difficult for the transcriptional machinery to access DNA, leading to an inhibition of gene transcription [20]. To date, 18 mammalian HDACs have been identified and categorized into four groups, as shown in Figure 1: class I HDACs (HDAC1, 2, 3, 8); class II HDACs (HDAC4, 5, 6, 7, 9, 10); class III (sirtuin family: sirt1-7); and class IV with a single member (HDAC11) [21]. Class II HDACs are further divided into two subgroups: Class IIa, which has a large C-terminal domain, and Class IIb, with two deacetylase domains. Class I, II, and IV HDACs are zinc-dependent enzymes. Class I HDACs are present in all cells and are homologous to Rpd3 in yeast, traditionally considered the nuclear histone deacetylases. Class I HDACs are closely associated with several other protein subunits, including Sin3 and N-CoR, with which they mediate histone deacetylation and transcriptional co-inhibition [22]. Class II HDACs are homologous to yeast HDA1, having an extended N-terminal and catalytic structural domain, and they exhibit increased expression in striated muscle and the brain [21]. Class IIa HDACs (4, 5, 7, 9) have the ability to shuttle between the nucleus and cytoplasm and are expressed in the brain, heart, and muscles [23]. Class IIb HDACs (6, 10) are mainly located in the cytoplasm [23], with one study indicating that HDAC6 is expressed in myotome [24]. Class III HDACs are niacinamide adenine dinucleotide (NAD^+^)-dependent enzymes [21] that are involved in many biological processes, including cellular metabolism, stress response, and aging [25]. SIRT stands for “silent mating type information regulator” and was originally identified and named as a gene silencer that controls mating type in yeast [26]. They are further subclassified based on sequence homology and subcellular localization: SIRT1 is mainly located in the nucleus and shuttles into the cytosol under certain conditions [27]. SIRT2 is primarily located in the cytoplasm but is also present in the nucleus during the G2-to-M phase transition of the cell cycle [28]. SIRT3-5 are mainly localized in the mitochondria due to the presence of mitochondrial targeting sequences [29]. SIRT6-7 are nuclear proteins. SIRT6 is localized in chromatin, while SIRT7 is mainly present in nucleoli. Finally, the only class IV HDAC11 has sequence homology with class I and II HDACs. Its functions involve maintaining the protein stability of DNA replicators CDT1 and IL10 [30], as well as regulating transcription when associated with bromodomain protein 2 [31]. HDACs are usually combined with other proteins to form macromolecular structures that act as corepressor complexes and play a role in the silencing of several genes related to processes such as survival, proliferation, cell growth, angiogenesis, and differentiation. HDACs play a crucial role in post-translational modifications associated with cardiovascular diseases, and the discovery of novel HDACi drugs may facilitate their treatment by modulating HDAC functions.

### 1.4. Histone Deacetylase Inhibitors (HDACis)

HDACis have been highlighted as anticancer drugs with unique effects of inducing growth arrest, differentiation, and apoptosis [32,33]. HDACis are a class of natural and synthetic compounds that promote histone acetylation and chromatin remodeling, facilitating proper nucleosome positioning and reverting gene expression [34]. In addition to histones, small-molecule HDACis have also been found to regulate the acetylation levels of non-histone proteins, which is potentially valuable for disease treatment [35]. Most zinc-dependent HDACs consist of three components: a zinc-binding group (ZBG) located in the active site of the HDAC, a recognition structure (the Cap structure) that interacts with the entry residues in the active site, and a linker structure that binds these two components [36]. Class III HDACs require NAD^+^ for intrinsic activity, whereas class I, II, and IV HDACs are zinc-dependent. Therefore, most class I and II HDACis target zinc ions. HDACis can be classified into four categories based on their chemical structure: short-chain fatty acids (such as sodium butyrate and valproic acid), cyclic peptides (such as Apicidin and Romidepsin), hydroxamic acids (such as Trichostatin A, Vorinostat, Belinostat, and Panobinostat), ad benzamides (such as Entinostat and Mocetinostat), as shown in Table 1. In recent years, many novel inhibitors targeting various metal-binding regions with monosulfonamide and thioacetyl groups have also been discovered. Hydroxamic acids are the most studied class of drugs with the highest molecular weight and short half-life [37], which can inhibit Class I and Class II HDACs with nM potency. Cyclic peptides are the most complex class of HDACis, including Apicidin, Romidepsin, and a group of peptides containing cyclic hydroxamic acids, which are generally considered to be Class I HDACis. Benzamide is an orally bioavailable drug that effectively and selectively inhibits class I and IV HDAC enzymes [38]. Short-chain fatty acids are relatively modest HDACis with some limited class I HDAC selectivity. Recent studies have proved that the active ingredients of Chinese herbs, such as ginsenosides, cinnamic acid [39], etc., also have HDAC inhibitory effects multiple studies have shown that HDACis are beneficial for cardiovascular disease, so it is important to develop a greater diversity of HDACis.

In this review, we systematically analyze the effects between the four classes of HDACs in post-translational modifications and common cardiovascular diseases. Additionally, we explore the regulatory mechanisms of HDACis in pathophysiologically relevant post-translational modifications, offering novel insights for future therapeutic strategies targeting cardiovascular diseases.

## 2. HDACs and Post-Translational Modifications Are Closely Associated with Cardiovascular Diseases

### 2.1. Cardiac Hypertrophy

Cardiac hypertrophy occurs when the heart adapts to continuously elevated blood pressure or continuously increased blood volume by increasing the heart muscle mass [40]. Based on its functional significance, cardiac hypertrophy can be categorized into physiological and pathological types, which are closely related to the nature, duration, and magnitude of the increased cardiac workload [41]. While physiological cardiac hypertrophy is associated with normal cardiac physiological processes, its pathological equivalent ultimately leads to heart failure and is characterized by an absolute increase in ventricular mass in response to different stressors [41].

Class I HDACs are often thought to play a role in promoting cardiac hypertrophy. There is evidence that HDAC1 and HDAC2 contribute to inhibit cardioprotection and anti-hypertrophy inhibition [36]. Calmodulin-dependent protein kinase II (CaMKII) is a direct downstream target of β-adrenergic [42] as well as Gαq signaling [43] (endothelin and angiotensin II), and mediates cardiomyocyte death [44], cardiac hypertrophy [45], as well as fibrosis [46] driven by neurohormone overactivity. Zhang et al. demonstrated that CaMKII can directly phosphorylate HDAC1, 2, and 3, to enhance their deacetylase activity in vitro, which may be related to their role in severe cardiac hypertrophy. HDAC2 is regulated by a variety of post-translational modifications, including serine phosphorylation, lysine ubiquitination, as well as tyrosine and cysteine nitration. Hypertrophy stimulators induce physical interaction between heat shock protein 70 (HSP70) and HDAC2, thereby selectively targeting HDAC2 in the heart [47]. In addition, when myocardial cells are infected with acetylated mimic mutants of HDAC2, the antihypertrophic effects of HDAC5 and leptomycin B-induced nuclear retention or overexpression of HDAC5 are significantly weakened. Hypertrophy stimulation promotes the translocation of casein kinase 2 (CK2) into the nucleus, resulting in the phosphorylation of HDAC2 on serines 394, 411, 422, 424, and S394 is the key site, which ultimately promotes the growth of cardiomyocytes [48]. In addition, in cardiac-specific HDAC3 knockout mice, HDAC3 cooperates with SMRT/n-CoR to a reduce histone acetylation in the vicinity of myocyte enhancer factor 2 (MEF2) [49], resulting in abnormal energy metabolism and cardiac hypertrophy [50]. Myofibril growth during cardiac hypertrophy is regulated by phosphorylation and acetylation of the actin-capping protein CapZβ1, and phenylephrine (PE) treatment of cardiomyocytes reduces HDAC3 binding to myofibrils, which leads to the phosphorylation of CapZβ1 at the serine 204 site and acetylation of CapZβ1 at the lysine 199 site [51]. The overexpression of HDAC8 can stimulate cardiac hypertrophy, promote the phosphorylation of p38 MAPK as well as the expression of atrial natriuretic peptide (ANP) and brain natriuretic peptide (BNP) proteins, and inhibit myocardial hypertrophy by blocking HDAC8 activity [52].

Class IIa HDACs are expressed in the heart. They can directly bind to other hypertrophy-promoting transcription factors to inhibit their target genes, including GATA binding protein 4 (GATA4) [53], MADS-box family member serum response factor (SRF) [54], MEF2 [55], and nuclear factor of activated T-cells (NFAT) [56]. HDAC IIa is generally regarded as a cardiac anti-hypertrophic molecule whose function depends on binding to MEF2 and subsequent inhibition [57]. MEF proteins are a family of myocyte-specific enhancers that are responsible for transcriptional regulation during cardiomyocyte development [58]. Under pathological conditions such as continuous β-adrenergic receptor stimulation, angiotensin II (Ang II) infusion, or stress overload, MEF can promote the transcriptional regulation of cardiac hypertrophy [59]. HDAC IIa is usually combined with MEF2C. When cardiomyocytes are stimulated by pathological stress, HDAC is transported out of the nucleus, where MEF2C recruits p300 to chromatin in the absence of HDAC II, thereby enhancing the transcription of hypertrophy-related genes. The HDAC IIa-MEF2 pathway plays an important role by inhibiting fetal gene transcription and preventing cardiac hypertrophy. The activity of the HDAC II-MEF2C complex is regulated by HDAC II phosphorylation. In addition, HDAC4 forms an inhibitory complex with SUV39H1 to maintain MEF2 in the H3 methylation state, thereby reducing the expression of ANP and BNP, resulting in cardiac protection [60]. Since SUV39H1 is a nuclear histone methyltransferase, this interaction reveals the crosstalk mechanism between histone deacetylation and histone methylation [61]. As a response to adverse stimuli, CaMKIIδB increases the phosphorylation of HDAC4 and its translocation from the nucleus to the cytoplasm, leading to a decrease in HDAC4 levels in the nucleus. This change causes the HDAC4/SUV39H1 complex to dissociate, triggering H3K9 demethylation, Mef2 transcriptional activation, and ultimately cardiac hypertrophy [61]. The N-terminal region of Class II HDACs contains two conserved CaMK phosphorylation sites [62]. Phosphorylation of Class II HDACs by CaMK and other kinases can break the close interaction with MEF2, decreasing transcriptional activity. HDAC5 and HDAC9 have similar functions as endogenous inhibitors of cardiac hypertrophy in vivo [55,63]. The mechanism by which their dysfunction promotes hypertrophy involves the phosphorylation of two conserved serine residues on either side of the nuclear localization signal (S259 and S498 in HDAC5 and S218 and S448 in HDAC9). Phosphorylation mutants of HDAC5 at serines 259 and 498 exhibit resistance to PKC-induced signaling and attenuated cardiac hypertrophy [64]. Phosphorylation promotes binding to the 14-3-3 protein, which shields the nuclear localization sequence and induces a conformational change that exposes the C-terminal nuclear output sequence for nuclear export via the chromosomal region maintenance 1 (CRM1) protein [65,66].

The mechanism of action of HDAC IIb has been discovered in recent years. Studies have shown that knockout of the HDAC5 and HDAC6 genes can block the COX2/PGE2 pathway in response to Ang II-induced cardiac hypertrophy. In addition, the HDAC inhibitor sodium butyrate (NaB) can effectively inhibit the expression of COX2/PGE2. Ang II increases the levels of ANP and phosphorylated ERK (pERK), whereas NaB reverses this effect both in vivo and in vitro [67]. In summary, Ang II can induce cardiac hypertrophy by triggering an HDAC5/HDAC6-dependent mechanism that can be reversed by NaB. However, until now, no specific studies have investigated the role of HDAC10 in cardiac hypertrophy.

The SIRT protein family plays an important role in maintaining cardiac homeostasis. Studies have shown that oxidative stress is a key metabolic characteristic of aging, whereby excessive ROS can trigger DNA damage and cell-cycle arrest [68], while SIRTs have cardioprotective functions that can effectively prevent oxidative damage and the development of age-related lesions [69]. During cardiac hypertrophy, the expression levels of SIRT1, SIRT3, and PGC-1α (peroxisome proliferator-activated receptor γ-coactivator 1α) are significantly reduced [70,71]. SIRT1 and SIRT3 protect cardiomyocytes by enhancing the deacetylation of PGC-1α, reducing oxidative stress, and inhibiting the occurrence of cardiac hypertrophy [72,73]. It has been shown that knockdown of SIRT1 increases the level of crotonylation of SERCA2a to decrease its activity, which in turn affects the expression of proteins in the PPAR pathway, leading to changes in energy metabolism and alterations in physiological states such as cardiac hypertrophy [74]. The knockdown of SIRT5 resulted in increased lysine succinylation of proteins in the heart, which exhibited defective fatty acid metabolism, reduced ATP production, and hypertrophic cardiomyopathy [75]. The knockout of SIRT3 induces the acetylation of long-chain acyl-CoA dehydrogenase at lysine 42 and blocks fatty acid oxidation, thereby downregulating ATP synthesis and other processes [76]. SIRT3 can also reduce the accumulation of hypertrophy-associated lipids in the heart through deacetylation of LCAD, thereby slowing the development of cardiac hypertrophy [77]. Reactive oxygen species (ROS) and oligomycin sensitivity-conferring proteins (OSCP) are associated with the acetylation of SIRT3 substrates, and both are key regulators of cardiac hypertrophy [78]. SIRT3 increases NADPH levels through the deacetylation of IDH2, thereby increasing GSH and decreasing ROS levels [79]. These results suggest that SIRT3 is an endogenous negative regulator of cardiac hypertrophy that protects the heart by reducing cardiomyocyte ROS levels. SIRT3 can deacetylate and thereby activate Mn-superoxide dismutase (MnSOD), which catalyzes the disproportionation of superoxide radicals. However, the overexpression of SIRT4 can inhibit the interaction of MnSOD with SIRT3, resulting in an increase in the acetylation level of MnSOD and a decrease in its activity, and ultimately promote angiotensin II-induced hypertrophic growth in mice [80]. In addition, SIRT2 [81] and SIRT6 [82] have also been shown to prevent cardiac hypertrophy, during which the IGF-AKT signaling pathway is continuously activated. It was found that SIRT6 acts as an endogenous negative regulator of this process in cardiomyocytes. SIRT6 deficiency leads to an elevated H3K9 acetylation level and enhanced interaction with the stress-responsive transcription factor c-Jun, further promoting IGF signaling and ultimately leading to cardiac hypertrophy [83]. It also protects the myocardium from hypertrophy by reducing the protein level of p300 and subsequently decreasing the acetylation and transcriptional activity of the NF-κB p65 subunit [84]. SIRT7 has been shown to play a role in cardiac hypertrophy and the associated reduced lifespan. According to Vakruhsheva et al., SIRT7 deacetylates p53, inactivating it and thus preventing apoptosis [85]. Nicotinamide mononucleotide adenylyltransferase is not only a key enzyme in the biosynthesis of NAD^+^, but is also closely related to the activation of SIRTs and has been shown to inhibit angiotensin II-induced cardiac hypertrophy [86]. Furthermore, studies have shown that in muscle cells, the activation of AMP-activated protein kinase (AMPK) increases intracellular NAD^+^ levels, enhances SIRT1-mediated protein deacetylation, and activates downstream targets such as PGC-1α and forkhead box protein O1 (FOXO1). At the same time, the AMPK/SNF1 pathway triggers histone acetylation by phosphorylating them and promoting the assembly of the HAT complex, which in turn enhances the transcriptional activity of specific genes [87]. In this context, the activation of SIRTs is closely related to the AMPK/SNF1 pathway, whereas AMPK inhibits the activation of HDAC IIa. Specifically, stronger AMPK activation leads to higher activation levels of SIRTs and more HDAC IIa being transferred to the cytoplasm, resulting in an inhibitory effect on cardiac hypertrophy.

**Figure 2:**
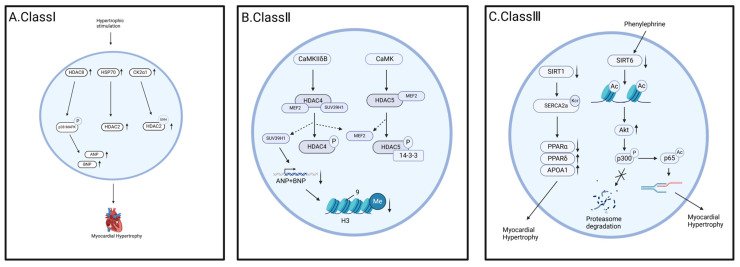
Regulatory mechanisms of post-translational modification of HDAC in cardiac hypertrophy. (**A**). Class I HDAC, when hypertrophy stimulation acts on cardiomyocytes, the expression of HDAC8, HSP70, and CK2α1 is up-regulated. HDAC8 promotes p38 MAPK phosphorylation and increases the expression of cardiac hypertrophic markers, such as ANP and BNP. HSP70 inhibits the expression of antihypertrophic genes by increasing the activity of HDAC2. CK2α1 increases the activity of HDAC2 by promoting the phosphorylation of HDAC2 S394 and promotes cardiac hypertrophy. (**B**). Class II HDAC, CaMKIIδB induces the dissociation of HDAC from SUV39H1 and MEF2, promotes the phosphorylation of HDAC, reduces the methylation of H3K9, inhibits the transcription of ANP and BNP genes, and thus results lead to the cardiac hypertrophy; CaMK can dissociate HDAC5 from the MEF2 complex, promote the phosphorylation of HDAC5 and the binding of HDAC5 to 14-3-3. (**C**). Class III HDAC, the reduction of SIRT1 promotes the crotonylation of SERCA2a, which affects the expression of proteins in the PPAR pathway and leads to disorders of energy metabolism; the induction of phenylephrine leads to the reduction of SIRT6 expression and deacetylase activity, which through transcriptional regulation leads to the elevation of Akt expression, which in turn promotes the phosphorylation of p300 and inhibits the degradation of ubiquitin-proteasome. It also leads to the acetylation of p65, a subunit of NF-κB, which has enhanced transcriptional activity and triggers a hypertrophic response.

Overall, HDACs and various associated post-translational modifications play important roles in cardiac hypertrophy, with phosphorylation being the most extensively studied. Post-translational modifications may regulate nuclear-cytoplasmic shuttling, such as the phosphorylation of HDAC-IIa by CaMKII under pathological stimulation, which releases the inhibition of MEF2 and allows the transcription of hypertrophic genes. In summary, it was found that various types of HDACs play different roles. Class IIa HDACs and Class III HDACs play a protective role, whereas some of Class I can promote cardiac hypertrophy, as shown in Figure 2. However, there are still many mechanisms that have not been elucidated and will require further research in the future.

### 2.2. Heart Failure

Heart failure (HF) is a disease caused by a blood-pumping disorder in the heart, resulting in systolic or diastolic dysfunction, followed by reduced circulation and insufficient arterial perfusion [88]. HF is the most common cause of death due to CVD, affecting an estimated 64.3 million people worldwide [89]. This is a very complex disease in which various molecules and cells undergo various changes during its onset and following chronic disease process [90]. These changes not only affect the heart’s structure but also impair its contractile function. Heart failure is characterized by various changes in the body, including alterations of cardiomyocyte apoptosis, the development of cardiac fibrosis, and altered gene expression. However, the underlying mechanisms are still not fully understood.

HDACs downregulate gene expression in heart failure by acting as transcriptional repressors. Methyltransferase-like protein 7B (METTL7B), a member of the methyltransferase-like family containing the methyltransferase structural domain, inhibits the expression of USP38 through m6A-dependent mRNA degradation, leading to increased ubiquitination of HDAC3, which maintains histone lactylation at a later stage to improve cardiac remodeling and thus prevent heart failure [91]. HDAC3 deacetylates DNA methyltransferase 1 (DNMT1) to inhibit ubiquitination-mediated degradation, which promotes the expression of DNMT1, which inhibits the expression of SHP-1 by methylation of the promoter region, thereby leading to heart failure induced by cardiomyocyte hypertrophy [92].

Class IIa histone deacetylases inhibit cardiomyocyte hypertrophy by binding to the hypertrophic transcription factor (TF) myocyte enhancer factor-2 (MEF-2). Class IIa HDAC4, 5, 7, and 9 are affected by signal-dependent phosphorylation of Ca^2+^/calmodulin-dependent protein kinase (CaMK) family members. The phosphorylation of HDAC7 by salt-inducible kinase 1 (SIK1), a member of the CaMK family, stabilizes the deacetylase, leading to an increase in c-Myc expression, which in turn promotes the progression of heart failure [93]. However, there is little research on class IIb HDACs in relationship to post-translational modifications. Previous studies have shown that HDAC6 is involved in cardiac hypertrophy and may also be involved in heart failure. HDAC10 interacts with thioredoxin to regulate the alteration of ROS signaling in cancer cells, so there may be many hidden mechanisms that we need to explore.

SIRTs play a beneficial role during the development of heart failure. SIRT1 plays an important role in the process of improving heart failure. For example, SIRT1 reduces ferroptosis and ameliorates heart failure by decreasing the K382 acetylation level of p53 and degradation of SLC7A11, while increasing GSH abundance and glutathione peroxidase 4 (GPX4) expression [94]. SIRT1 also increases the deacetylation of FOXO3a, which in turn prevents apoptosis and ameliorates heart failure [95]. SIRT1 was found to improve cardiac function, reduce ventricular mass, and decrease apoptosis in cardiomyocytes by inhibiting the acetylation of NF-κB p65, leading to a decrease in the expression of NF-κB p65, which ameliorates heart failure [96]. SIRT1 significantly protected cardiomyocytes by decreasing miR-138-5p by increasing the deacetylation level of p53 [97]. In clinical practice, low expression of SIRT1 may upregulate antioxidants and pro-apoptotic molecules by increasing p53 acetylation and decreasing FOXO1 translocation into the nucleus [98]. Gorski et al. reported that SIRT1 controls cardiac SERCA2a function by acetylating cardiac SERCA2a at K492, which is critical for the pharmacological activation of SIRT1-mediated deacetylation and normalization of SERCA2a activity [99]. SIRT3 activation and mitochondrial metabolic processes are mediated by acetylation and SUMO crosstalk, but more crosstalk still needs further exploration [100]. Transforming growth factor β-1 (TGF-β1) mediates the transformation of fibroblasts into myofibroblasts, regulates tissue fibrosis, and increases the synthesis of extracellular matrix components. The loss of SIRT3 leads to the expression of TGF-β1 and hyperacetylation of glycogen synthase kinase 3β (GSK3β) at Lys15, while the deacetylation of SIRT3 simultaneously activates GSK3β, thereby reducing the fibrosis of cardiac tissue [101]. SIRT3 also regulates ferroptosis and cardiac fibrosis by regulating the acetylation of p53 [102]. SIRT3 deficiency leads to mitochondrial protein hyperacetylation and mitochondrial dysfunction. For example, downregulated SIRT3 expression in the myocardium of HF patients with obesity and metabolic syndrome leads to cyclophilin D (CypD) hyperacetylation, mitochondrial permeability transition pore (mPTP) opening, and cardiac insufficiency [103]. Therefore, SIRT3 is a key regulator of cardiac energy metabolism and a potential mitochondrial target for targeted drugs. In addition, SIRT5 can reduce isocitrate dehydrogenase 2 (IDH2) succinylation levels, protect cell viability, maintain mitochondrial homeostasis, and ameliorate myocardial fibrosis, thereby reducing the incidence of heart failure [104].

HDAC11 has been identified as a negative regulator of the well-known anti-inflammatory cytokine IL-10 [105]. This cytokine is associated with active inflammation in the early stages of heart failure through association with DNA methylation, which downregulates IL-10 [106]. Further research is needed to explore the relationship between HDAC11 and HF.

**Figure 3:**
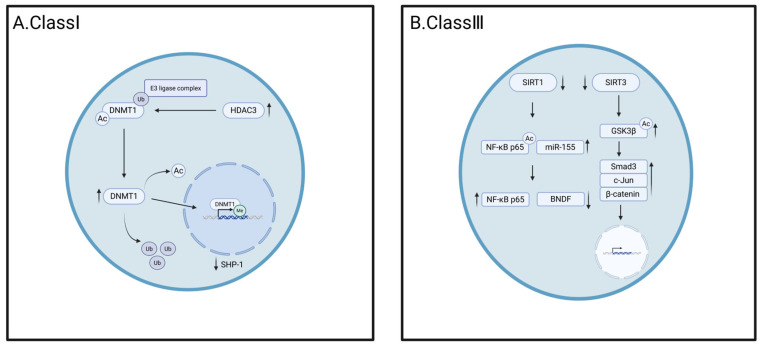
Regulatory mechanisms of post-translational modification of HDAC in heart failure. (**A**). Class I HDAC, HDAC3 is highly expressed in the cardiomyocyte hypertrophy model, which causes DNMT1 deacetylation and inhibits ubiquitination mediated proteome degradation, promotes DNMT1 expression, enters the nucleus and methylates SHP-1 promoter region, down-regulates SHP-1 expression, and leads to heart failure; (**B**). Class II HDAC, SIRT1 attenuated induced NF-κB expression and downregulated miR-155, which in turn inhibited BNDF expression; SIRT3 expression was downregulated, GSK3β acetylation was enhanced, and its phosphorylation was inhibited, which in turn led to the increased expression of Smad3, c-Jun, and β-catenin, and entry into the nucleus to regulate the expression of pro-fibrotic genes.

The study of HDACs in heart failure is currently limited, especially in the context of various post-translational modifications, as shown in Figure 3. Various histone deacetylases (HDACs) regulated by post-translational modifications (PTMs) perform distinct functions by targeting different substrates. For instance, SIRT1 confers cardioprotection through the deacetylation of p53 within the nucleus, whereas HDAC3 facilitates heart failure by deacetylating DNMT1 in the nucleus. This area needs to be explored in depth in the future, and related studies are expected to reveal potential new directions for the treatment of heart failure.

### 2.3. Atherosclerosis

Atherosclerosis (AS) is characterized by the yellowish color of the lipids accumulated in the arterial lining and is one of the most common types of cardiometabolic disease [107]. Atherosclerosis plays a key role in the pathogenesis and progression of multiple cardiovascular diseases, including coronary and peripheral artery disease [108]. Its main feature is the thickening of arterial walls due to the formation of plaques under the endoderm, composed of fatty acids, cholesterol, calcium, fibrin, cell debris, and metabolic waste. These lesions can lead to varying degrees of narrowing of the arteries or even complete blockage of blood flow, leading to a lack of oxygen to the heart, brain, kidneys, pelvis, arms, or lower limbs [109]. As the plaque grows, it may become less stable and rupture, leading to the formation of a localized blood clot that can further block downstream veins or arteries. This condition is commonly referred to as thrombosis [110]. In severe cases, this can further lead to life-threatening complications such as myocardial infarction (MI) and stroke.

HDAC1-3 play different roles in various regulatory processes related to atherosclerosis. Increased expression of the vascular cell adhesion molecule VCAM-1 on the surface of activated arterial endothelial cells (EC) is closely related to the occurrence of atherosclerosis. Hu et al. demonstrated that HDAC1 and HDAC2 can further reduce the methylation of the GATA6 promoter by inhibiting the acetylation of STAT3 at lys685, thereby regulating the expression of VCAM-1 in endothelial cells and promoting the formation of atherosclerotic lesions [111]. Endogenously expressed microRNAs (miRNAs), small non-coding RNAs that regulate gene expression at the post-transcriptional level [112], have been demonstrated to play a pivotal role in diverse biological and cellular processes [113]. For example, miR-21, miR-23a, and miR-24 have been implicated in the regulation of the development of cardiac hypertrophy [114]. Recent studies have shown that miRNAs are significantly dysregulated in the pathological processes of atherosclerosis and cardiovascular diseases, involving multiple aspects, such as endothelial cell activity, macrophage function, and vascular smooth muscle cell behavior [115,116,117]. Wang et al. found that the overexpression of HDAC1 enhanced miR-224-3p-mediated FOSL2 inhibition and inhibited the progression of atherosclerosis by deacetylating HIF-1α [118]. Zhao et al. further explored how hyperhomocysteinemia (HHcy) promotes atherogenesis by altering histone acetylation patterns and regulating miRNA expression. Studies have shown that Hcy-induced atherosclerosis is mediated by the increased expression of HDAC1, and the upregulation of HDAC1 reduces the level of acetylation of histone H3 at the lys9 (H3K9ac) site, thereby inhibiting the expression of miR-34a. This in turn contributes to the accumulation of total cholesterol (TC), free cholesterol, and triglycerides, accelerating the process of atherosclerosis [119]. Matrix metalloproteinase-9 (MMP-9) is a zinc-dependent endopeptidase capable of degrading extracellular matrix components, and its increased expression is closely related to the occurrence of atherosclerosis. Interferon-β (IFN-β) can somewhat inhibit the progression of atherosclerosis by downregulating the mRNA expression of MMP-9. In the specific mechanism, IFN-β reduces the acetylation level of histone H3 by increasing the recruitment of HDAC-1 to the MMP-9 promoter region, thus inhibiting its transcriptional activity. The results also indicated that the proximal AP-1 site plays a key role in this inhibitory process. When the AP-1 site is inactivated by a point mutation, the IFN-β-mediated transcriptional inhibition effect disappears, thereby promoting the further development of atherosclerosis [120]. Macrophage-triggered chronic inflammation and smooth muscle cell-induced vascular remodeling are two main pathophysiological processes in the formation of atherosclerosis. The main histocompatibility class II (MHC II) transactivator (CIITA) is a transcriptional regulator of MHC II activation and type I collagen inhibition induced by interferon-γ (IFN-γ). CIITA and HDAC2 interact inside smooth muscle cells and macrophages. HDAC2 can reduce the interaction between CIITA and RFX5 by protein degradation and deacetylation of CIITA, thereby counteracting the activity of CIITA and promoting the development of atherosclerosis [121]. In another study, HDAC2 was shown to protect against atherosclerosis. In male mice, when HDAC2 is absent, there is an increase in acetylation on histone 3, an increase in GRX1 expression, as well as an association with increased MKP-1 activity and a decrease in monocyte derivation and macrophage recruitment [122]. P21 plays a pivotal role in vascular remodeling. The phosphorylation of HDAC2 facilitates its dissociation from RARα and enhances its interaction with KLF transcription factor 5 (Klf5), resulting in the deacetylation of Klf5. This process subsequently promotes the dissociation of Klf5 from the p21 promoter, thereby diminishing the inhibitory effect on the p21 promoter and ultimately contributing to the progression of atherosclerosis [123]. Vascular smooth muscle cell (VSMC) aging plays a critical role in the development of atherosclerosis. VSMC-specific TRAP1 deficiency alleviates VSMC aging and atherosclerosis via metabolic reprogramming. Conversely, TRAP1 significantly increased aerobic glycolysis, leading to elevated lactate production. Accumulated lactate promoted histone H4 lysine 12 lactylation (H4K12la) by down-regulating the unique histone lysine delactylase HDAC3, promoting senescence-associated secretory phenotype (SASP) expression. These findings may offer a novel therapeutic strategy for atherosclerosis [124].

Among enzymes of the HDAC IIa subtype, HDAC4 is capable of inhibiting the expression of miR-148b-3p and promoting the transcription of NCOR1 by reducing the levels of H3 and H4 acetylation in the promoter region of the miR-148b-3p/NCOR1 gene cluster. This process further promotes the overexpression of KLF7, which has a protective effect on the cells and effectively reduces the incidence of atherosclerotic lesions [125]. Betulinic acid (BA) is a naturally occurring pentacyclic triterpenoid compound with anti-inflammatory, metabolic modulatory, and cardiovascular protective properties. BA can upregulate the expression of the transcription factor KLF2, increase the intracellular Ca^2+^ level, activate the CaMKKβ, CaMKIIα, and AMPK signaling pathways, enhance the phosphorylation of ERK5, HDAC5, and MEF2C, and ultimately induce the expression of eNOS, thereby exerting a preventive effect against atherosclerosis [126]. By contrast, among the HDAC IIb isoforms, lncRNA NORAD enhances the deacetylation of H3K9 by recruiting HDAC6 to the promoter region of the VEGF gene, thereby inhibiting its transcription, leading to increased vascular endothelial cell injury and promoting the development of atherosclerosis [127].

SIRT1 plays a protective role in the development and progression of atherosclerosis [128]. Senescence leads to atherosclerosis, and LA ribonucleoprotein 7 (LARP7) is a senescence antagonist. When DNA damage-mediated activation of the ataxia telangiectasia mutated (ATM) gene triggers extracellular shuttling and the downregulation of LARP7, it inhibits the activity of SIRT1 deacetylase. This enhances the acetylation of p53 and NF-κB (p65) to increase their transcriptional activity, accelerating cellular aging and atherosclerotic progression [129]. S-adenosyl homocysteine (SAH) is a risk factor for cardiovascular disease. The inhibition of SAH hydrolase (SAHH) leads to the accumulation of SAH and the inhibition of DNMT3b (DNA methyltransferase 3b), which results in hypomethylation of the H19 promoter and reduced intracellular adenosine levels, combined with reduced activation of AMPK (AMP-activated protein kinase), which in turn inhibits SIRT1 mediated-hyperacetylation of histone H3 to promote atherosclerosis [130]. Long non-coding RNA (lincRNA) p21 is involved in the development of atherosclerosis, and competitively binds to miR-221 to promote the deacetylation of Pcsk9 by SIRT1, thereby reducing the progression of atherosclerosis [131]. It has been shown that senescent endothelial cells have low levels of nitric oxide synthase (eNO), while nitric oxide (NO) has vasodilator, antioxidant, and atheroprotective effects [132]. Mattagajasingh et al. showed that SIRT1 activates eNO during caloric restriction by increasing endothelial nitric oxide synthase levels through deacetylation. By contrast, decreased levels of SIRT1 lead to increased acetylation of eNO on lysine residues 496 and 506, inhibiting its activity [133]. Amp-activated protein kinase (AMPK)-mediated phosphorylation, SIRT-1-mediated coronin deacetylation, and SIRT-1-mediated eNOS deacetylation have atheroprotective effects in human umbilical vein endothelial cells (HUVECs) in vitro. At the same time, eNOS deacetylation of HDAC3 at K610 promotes atherosclerosis [133,134,135]. Jiang et al. showed that SIRT2 stabilizes the gluconeogenesis process during fasting through the deacetylation of phosphoenolpyruvate carboxykinase (PEPCK1), which inhibits its ubiquitination and degradation, thereby activating and maintaining its function in gluconeogenesis [136]. N ε-carboxymethyl lysine (CML), an active component of advanced glycation end-products (AGEs), significantly enhances the activity of nuclear factor of activated T-cells 1 (NFATc1) by mediating crosstalk between acetylation and phosphorylation. This process leads to the downregulation of SIRT3, increased acetylation of NFATc1 at lysine 549 (K549), and antagonizes the phosphorylation of NFATc1 at tyrosine 270 (Y270) by focal adhesion kinase (FAK). Consequently, these mechanisms promote the progression of atherosclerosis [137]. ROS play a key role in atherosclerosis, and SIRT3 can reduce oxidative stress by reducing mitochondrial ROS production through epigenetic regulation. SIRT3 deacetylates forkhead transcription factor O subfamily member 3a (FOXO3a) and protects mitochondria from oxidative stress, further exercising protective antioxidant properties [138]. SIRT6 is an atheroprotective factor that mediates the deubiquitination of the K37 and K532 sites of HIF-1α [139]. SIRT6 is the target of miR-92a-3p, which activates the MAPK signaling pathway in vitro by negatively regulating SIRT6 as well as increasing the apoptosis and phosphorylation levels of JNK and p38 MAPK, which in turn exerts an anti-atherosclerotic effect [140]. Oxidized low-density lipoprotein (oxLDL) is a known risk factor for atherosclerosis, and cardiac myosin-related transcription factor A (MRTF-A) induces ICAM-1 transcription in response to oxLDL. Furthermore, SIRT6 interacts with MRTF-A to regulate the acetylation of MRTF-A, and promotes the deacetylation of MRTF-A to phosphorylate serine 154, mediating the inhibition of SIRT6 [141]. In addition, SIRT6 can also interact with apoptosis-associated speck-like protein (ASC) to inhibit its acetylation, thereby reducing the interaction between ASC and NLRP3 and inhibiting the apoptosis of endothelial cells, thereby slowing the development of atherosclerosis [142]. Kawahara et al. showed that SIRT6 alters NF-κB expression through the deacetylation of histone H3K9, thereby preventing the binding of the NF-κB RELA (REL-associated protein) subunit to its target gene promoter, reducing inflammation [143]. The combination of inflammation, cellular aging, and high LDL cholesterol constitutes a vicious cycle of atherosclerosis, and SIRT6 may stop this cycle. SIRT7-mediated succinylation of protein arginine methyltransferase 5 (PRMT5) enhances its activity and induces arginine methylation of SREBP1a, which promotes the biogenesis of fatty acids, TAGs, and cholesterol, which is strongly associated with atherosclerosis [144]. This is because HDACs are involved in regulating a wide range of biological processes, such as endothelial cells, smooth muscle cells, cholesterol metabolism, and inflammation. Arteriosclerosis can be better combated by developing HDACis that target the catalytic domain of HDAC [145].

**Figure 4:**
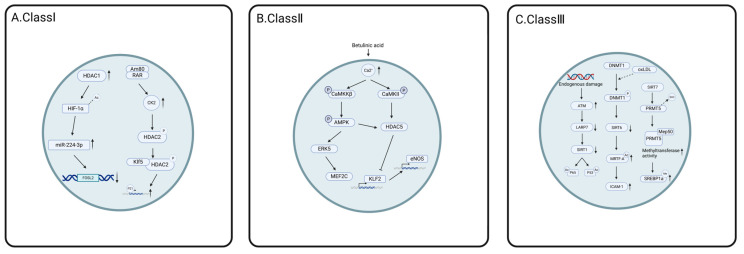
Regulatory mechanisms of post-translational modification of HDAC in atherosclerosis. (**A**). Class I HDAC, the upregulation of HDAC1 promotes deacetylation of HIF-1α, which facilitates miR-224-3p-mediated inhibition of FOSL2 and inhibits atherosclerosis; upon stimulation with Am80, CK2α expression is upregulated, leading to its translocation into the nucleus, where it phosphorylates HDAC2 and interacts with Klf5, which is essential for Klf5 deacetylation. Deacetylated Klf5 dissociates from the p21 promoter, thereby increasing p21 expression. (**B**). Class II HDAC, BA increased intracellular Ca2^+^ levels and activated the phosphorylation of CaMKKβ, CaMKII, and AMPK, leading to increased phosphorylation of HDAC5 and ERK5, which induced eNOS expression through the MEF2C pathway by increasing KLF2 transcriptional activity; (**C**). Class III HDAC and DNA damage-induced ATM activation result in the downregulation of LARP7, thereby inhibiting SIRT1 activity and enhancing the acetylation of both p53 and p65. Upon stimulation with ox-LDL, DNMT1 undergoes phosphorylation, which suppresses SIRT6 activity, promotes MRTF-A acetylation, and subsequently alleviates the inhibition of MRTF-A on the ICAM-1 promoter. Additionally, SIRT7 mediates the desuccinylation of PRMT5, facilitating the formation of the PRMT5-Mep50 complex, inducing methylation of SREBP1a, and contributing to the progression of atherosclerosis.

Consistent with the roles of various classes of HDACs in cardiac hypertrophy, each class of HDACs has its own role, with the most studied being acetylation and phosphorylation. Under acetylation, the same HDAC has different functions in different pathways. HDAC1 can enhance the expression of miR-224-3p and inhibit atherosclerosis, but it can also inhibit the expression of miR-34a and promote the development of atherosclerosis. The roles of various HDACs in atherosclerosis have received much attention, with HDAC1 and SIRT1 being the most studied, one promoting and the other inhibiting atherosclerosis, as shown in Figure 4. Various other HDACs may also have promoting or inhibiting effects on atherosclerosis, and the relevant mechanisms need to be further explored in the future.

### 2.4. Myocardial Ischemia/Reperfusion (I/R) Injury

I/R is a pathological process associated with a variety of diseases that can lead to cell death and organ damage. Ischemia arises when blood supply is restricted, leading to severe tissue hypoxia. Although restoring blood flow to ischemic tissues aims to alleviate ischemia, it may paradoxically induce further damage, a condition referred to as I/R injury. Myocardial I/R damage is widespread in ischemic heart disease, the leading cause of which is atherosclerosis [146]. When an atherosclerotic plaque becomes unstable and ruptures or erodes, it can trigger thrombosis, which may either cause the occlusion of the coronary artery lumen at the site of the plaque, or lead to distal coronary artery embolism [147]. In cases of acute myocardial infarction, early and successful revascularization can effectively prevent the loss of contractile myocardial mass, minimize infarct size, and enhance the prognosis [148]. Nevertheless, reperfusion may paradoxically result in increased and accelerated myocardial damage, a phenomenon referred to as myocardial I/R injury.

Class I HDACs play a protective role in I/R. Ischemic postprocessing (IPostC) has been proposed to reduce the risk of I/R injury. Autophagy is involved in I/R-induced myocardial injury in the elderly. By decreasing the binding of DNMT3b and HDAC2 to its promoter, DNA hypomethylation and H3K14 hyperacetylation of the miR-181a-2-3p promoter can be induced. This subsequently inhibits the binding of c-Myc to the miR-181a-2-3p promoter, ultimately alleviating I/R-induced myocardial injury [149]. GSK-3 is a serine/threonine kinase initially characterized as an enzyme capable of phosphorylating and downregulating glycogen synthase, which serves as the rate-limiting enzyme of glycogen metabolism. In addition, GSK-3β is also involved in I/R injury. HDAC3 regulates Ser9 phosphorylation of GSK-3β and thereby protects cells. The disruption of HDAC4 sumoylation resulted in HDAC4 accumulation in cardiomyocytes and impairment of HDAC4 ubiquitination, leading to a significant reduction in ROS levels. Therefore, SUMOylation of HDAC4 may be a prospective target for I/R therapy. HDAC6 levels are positively correlated with ROS production, while it protects against I/R injury by modulating the acetylation level of the antioxidant protein peroxiredoxin-1 (Prdx1) at the K197 site [150].

SIRT family members also play a protective role in myocardial tissue. SIRT1 enhances the transcriptional activity of forkhead protein O1 (FOXO1) by decreasing its acetylation levels, thereby upregulating the expression of ferritin heavy chain 1 (Fth1). As an inhibitor of ferroptosis, Fth1 suppresses the iron-dependent cell death of cardiomyocytes, safeguarding cardiac function against I/R injury [151]. The upregulation of SIRT1 and SIRT3 protein levels was found to result in decreased acetylation of p53 and PGC-1α, reduced phosphorylation of FOXO-1, elevated Bcl-2 levels and activity, as well as increased MnSOD levels. These changes collectively inhibit cardiomyocyte apoptosis and protect against I/R injury [152]. SIRT1 can regulate Nrf2 deacetylation, thereby upregulating the downstream signaling pathways of Nrf2 and enhancing its activity to protect cells. SIRT1 can regulate Nrf2 deacetylation, thereby upregulating the downstream signaling pathways of Nrf2 and enhancing its activity to protect cells [153]. In addition, SIRT1 prevents the phosphorylation of Akt and mitigates the reduction of Drp1 activation caused by anoxia/reoxygenation (A/R) injury, thereby inhibiting ischemia/reperfusion (I/R) damage [154]. By inhibiting hypoxia/reoxygenation (H/R) injury and modulating the phosphorylation levels of PI3K, AKT, and mTOR, it exerts protective effects against H/R-induced cardiomyocyte apoptosis and excessive autophagy [155]. SIRT1 deacetylates the p65 subunit of NF-κB at the lysine 310 site and blocks its transcriptional activity, thereby inhibiting oxidative stress and inflammatory responses. Isocitrate dehydrogenase 2 (IDH2) inhibits caspase-3 activation and alleviates mitochondrial damage via a SIRT3-dependent mechanism. In the IDH2 K413R mutant, this leads to enhanced enzymatic activity, decreased production of mitochondrial ROS, and mitigation of I/R injury [156]. Deficiencies in SIRT1 and SIRT3 result in the inactivation of AMPK and significant alterations of mitochondrial oxidative phosphorylation (OXPHOS), thereby impairing mitochondrial respiration under I/R stress conditions [157]. SIRT3 can inhibit reperfusion injury through the deacetylation of procyclin D, which prevents the opening of the mitochondrial permeability transition pore and subsequent cell death [158]. Sirtuin 5 (SIRT5) is a mitochondrial NAD^+^-dependent deacetylase that catalyzes the removal of succinyl groups from proteins. Proteomic analysis showed that the succinylation level of SIRT5 knockout mice was significantly higher than that of wild-type mice. The pretreatment of SIRT5 knockout mouse hearts with competitive succinate dehydrogenase inhibitors inhibited mitochondrial succinylation and mitigated I/R injury. This indicates that changes in succinate dehydrogenase activity significantly influence I/R damage, and succinylation may be a critical factor in the pathophysiology of I/R injury [159,160]. The loss of SIRT5 activity alters cardiac metabolic pathways, increases the succinylation of DHSA, promotes SDH activity, and exacerbates ischemia/reperfusion injury [161]. Charged multivesicular body protein 2B (CHMP2B), a subunit of the ESCRT-III complex, accumulates in the heart and impairs autophagic fluxes, while SIRT6 reduces the acetylation of FOXO1, promotes its transcriptional effect on the muscle-specific ubiquitin ligase Atrogin-1, and subsequently enhances the degradation of CHMP2B by Atrogin-1 to reduce I/R injury [162].

**Figure 5:**
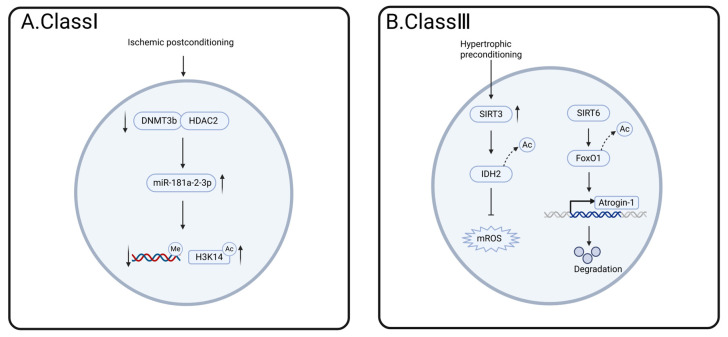
Regulatory mechanism of post-translational modification of HDAC in myocardial ischemia/reperfusion (I/R) injury. (**A**). Class I HDAC, IPostC treatment decreases the binding of DNMT3b and HDAC2 at the promoter region, thereby enhancing the expression of miR-181a-2-3p, promoting DNA hypomethylation and H3K14 hyperacetylation, and ultimately attenuating the effects of I/R injury. (**B**). Class III HDAC and TAC treatment upregulated the expression of SIRT3, leading to the deacetylation of IDH2, a reduction in mitochondrial ROS production, and improvement in I/R injury. In elderly cardiomyocytes, elevated SIRT6 levels decreased the acetylation of FoxO1, thereby enhancing its transcriptional activity on Atrogin-1 and promoting the degradation of CHMP2B.

Among the related post-translational modifications, the most extensive studies related to I/R injury have been conducted on class III HDACs, as shown in Figure 5. HDACs may play an important role in mitigating myocardial I/R injury by modulating key biological processes, such as oxidative stress and apoptosis. However, the molecular mechanisms behind these processes remain to be explored in depth.

### 2.5. Other Diseases

It has been found that SIRT3 deacetylase can effectively reduce inflammation and the pro-fibrotic response in the human heart and neonatal rat cardiomyocytes [163]. K27 acetylation of histone H3 on the promoter DNA promotes FOS transcription through the FOS/AP-1 pathway, thereby driving myocardial cell fibrosis and inflammation. However, SIRT3 can inhibit cardiac hypertrophy by participating in the deacetylation of histone H3 [164,165]. Acetylation plays a crucial role in the occurrence and development of atrial fibrillation (AF). Both in vitro and in vivo studies have shown that the expression of SIRT3 is significantly downregulated in AF, accompanied by the abnormal expression of key downstream metabolic factors. AF increases the acetylation levels of long-chain acyl-CoA dehydrogenase, Acetyl-CoA synthetase 2 (AceCS2), and Glutamate dehydrogenase (GDH), attenuating their enzymatic activities. This results in abnormal metabolic changes and a reduction in ATP levels. Therefore, SIRT3 is a critical factor affecting the acetylation status of key metabolic enzymes in atrial muscle. Enhancing SIRT3 expression can reverse the metabolic remodeling of atrial muscle induced by atrial fibrillation [166]. In addition, SIRT7 inhibits apoptosis by deacetylating p53 and enhancing stress resistance of cardiomyocytes in vitro, whereas Sirt7^−/−^ mice exhibited increased apoptotic signaling and an inflammatory cardiomyopathy phenotype in the heart [85]. At the same time, SIRT1 inhibits mitochondrial division and reduces the angiotensin II-induced apoptosis of cultured cardiomyocytes by deacetylating p53 [167]. Hyperlipidemia promotes the development of vascular injury by mediating oxidative damage through the activation of the adaptor protein p66, which interacts with cytochrome c to promote mitochondrial ROS production [168], proved to be a key regulator in cardiovascular disease [169]. SIRT1 can promote H3 deacetylation to inhibit p66 expression [170] and inhibit p66 function through K81 deacetylation [171], thereby protecting cells. HDACs mediate various post-translational modifications that play a key role in cardiovascular disease. Nevertheless, these findings still need to be validated by clinical evidence. With the development of new technologies in recent years, we can better understand and study the mechanism of HDACs in PTM in CVD. However, more evidence is needed to fully understand the roles of HDACs in CVD, which will provide new ideas and targets for the prevention and treatment of cardiovascular diseases.

## 3. Regulation of Cardiovascular Disease by HDACis Targeting Post-Translational Protein Acetylation

At present, a total of five HDAC inhibitors have been approved worldwide. Vorinostat, Romidepsin, Belinostat, and Panobinostat have been approved by the US FDA for the clinical treatment of peripheral T-cell lymphoma (Vorinostat, Belinostat), cutaneous T-cell lymphoma (Romidepsin), and multiple myeloma (Panobinostat). In addition, Chidamide (trade name: Epidaza^®^) has been approved by China’s NMPA for the treatment of peripheral T-cell lymphoma and breast cancer. Among these, Chidamide stands out as a selective inhibitor of HDAC Class I and the HDAC10 subtype, whereas the other four compounds act as pan-HDAC inhibitors. While inhibiting Class I HDACs and Class IIb HDACs, they also exhibit inhibitory activity against Class IIa HDACs (e.g., HDAC5, HDAC9) and Class IV HDACs (e.g., HDAC11), potentially leading to more toxic side effects mediated by the inhibition of Class IIa and Class IV HDAC enzymes.

Since the studies of post-translational modifications in drug development have mainly focused on the acetylation process, and the other modifications are less studied, the primary focus is on HDACis targeting protein acetylation. HDACs removes the acetyl group from lysine through a hydrolysis reaction. According to different catalytic mechanisms, the 18 types of HDACs can be divided into two categories, one comprising HDAC 1-11, which are zinc-dependent metalloenzymes, and the other comprising the seven sirtuins (SIRT1-7), which use NAD^+^ as a cofactor. The acyl group is transferred to the C2 position of the ribose sugar ring. Although these two enzyme families each perform specific biological functions in the process of hydrolyzing acyl lysine, HDACs are generally considered zinc-dependent enzymes. HDACis are currently one of the most widely studied types of drugs. Numerous studies have shown that HDACis can be used for cancer treatment. In addition to their role in tumor biology, HDACs play an important regulatory role in the pathological processes of multiple organs, including those affecting the nervous and cardiovascular systems [172]. In the cardiovascular field, it has been shown that some HDAC inhibitors, such as Trichostatin A (TSA), Vorinostat (SAHA), and valproic acid (VPA), have specific protective effects against cardiac hypertrophy, heart failure, atherosclerosis, and ischemia/reperfusion injury, as shown in Table 2. Protein acetylation has been known for over half a century, but its precise detection has only become available in the last two decades due to technical limitations. In addition, due to the technical and ethical issues of measuring acetylation levels in human patients receiving treatment, most studies have been conducted in animal models. Although these drugs have shown significant benefits in preclinical models, their clinical trials are still ongoing. Hence, these drugs require further study before they become viable options for clinical therapeutic interventions. To further improve the effectiveness and safety of HDACis and make up for the lack of specificity at this stage, more innovative strategies must be explored to optimize their design and application.

## 4. Directions for Future Research

Current studies on the roles of HDACs in the regulation of post-translational modifications mainly focus on acetylation and phosphorylation. Some studies have shown that HDAC1 undergoes SUMOylation at its C-terminus, which plays an important regulatory role in its transcriptional inhibition activity [189]. However, it is unclear whether SUMO directly alters the intrinsic activity of HDAC1 or whether it acts by regulating the formation of corepressor complexes. In addition, the Lys462 site of HDAC2 is also susceptible to SUMOylation, and SUMO-HDAC2 is catalytically active [190]. For example, Lys320 of p53 is targeted by SUMO-HDAC2, and deacetylated p53 cannot effectively regulate apoptosis of DNA-damaged cells [190]. S-nitrosylation is another important PTM of HDAC2, which was first discovered to play a role in neuronal development [191]. Nott et al. demonstrated that S-nitrosylation predominantly occurs at the Cys262 and Cys274 residues, it does not overlap with the phosphorylation site, and is a post-translational modification that directly acts on cysteine residues without relying on other modifications, which modulate HDAC2’s ability to bind to chromatin without altering its enzymatic activity [192]. Colussi et al. further investigated the therapeutic potential of HDAC2 S-nitrosation in a model of Duchenne muscular dystrophy. In this study, the intrinsic activity of HDAC2 was found to be significantly inhibited by S-nitrosylation, while endothelial nitric oxide synthase produced sufficient nitric oxide to inhibit HDAC2 activity. In addition, Malhotra et al. demonstrated HDAC2 S-nitrosylation and its functional relevance, which explains the inability to control chronic inflammation in chronic obstructive pulmonary disease (COPD) patients despite glucocorticoid therapy. S-nitrosylation can directly inhibit the activity of HDAC2, while de-nitrosylation can restore it. In summary, S-nitrosylation is an independent mechanism that regulates the intrinsic activity of HDAC2 and can reshape the transcription profile of its target genes. However, the functional role and biological significance of SUMOylation and S-nitrosylation of HDACs in CVD remain to be further elucidated. Therefore, in addition to studying the roles of HDACs in acetylation and phosphorylation, many other types of post-translational modifications are also relevant for this class of enzymes.

Post-translational modification is an indispensable mechanism of gene regulation that can rapidly respond to various cellular signals. Due to recent advances in high-resolution mass spectrometry and proteomics technologies, post-translational modifications have been extensively studied, revealing complex interactions between various modification types. When multiple PTMs target the same lysine residue, the underlying biological processes can be regulated through crosstalk or synergistic interactions [193]. For example, phosphorylation has been shown to affect protein acetylation [194,195,196]. Acetylation can, in turn, regulate protein phosphorylation, and HDACis targeting acetylation have also been found to affect protein phosphorylation. As an example, Class I HDACs inhibit phosphatase gene expression under stress conditions, whereas treatment with corresponding HDACis induces the expression of dual-specificity phosphatase 5 (dusp5) and further promotes dephosphorylation of extracellular signal-regulated kinase 1/2 (ERK1/2) by altering histone acetylation levels. The same study found that Class I HDACis can alleviate stress-induced cardiac hypertrophy through a mechanism that partially relies on phosphatase-mediated intracellular ERK1/2 dephosphorylation [197]. Class IIa HDACs are highly sensitive to cellular signals because there are multiple phosphorylation sites within their important regulatory regions (including the NES and NLS). In addition to phosphorylation, modifying serine and threonine residues with an O-linked β-N-acetylglucosamine (O-GlcNAc) group represents another dynamic and irreversible PTM. Studies have shown that, in addition to targeting the identical residues, phosphorylation and O-GlcNAcylation can also undergo crosstalk through adjacent interactions [198]. Therefore, studying the effect of HDACis on the crosstalk between post-translational modifications, such as acetylation–crotonylation, may provide new ideas for treating cardiovascular diseases.

In recent years, the emergence of single-cell technologies, including genomics, has provided people with more accurate biomedical data and met the needs of precision medicine [199]. The two main technologies involved in single-cell genomics are single-cell whole-genome DNA sequencing and single-cell RNA sequencing (scRNA-seq) [199]. Other single-cell level technologies include single-cell epigenetic sequencing, proteomics, and metabolomics. Each technique has its specific features, involving different views of genetic variation and mutation, DNA methylation, histone modification, transcription, translation, and metabolism, all of which are conducive to CVD research. Using single-cell genomics technology, new therapeutic targets have been identified for various cardiovascular diseases. Previous studies have found that scRNA-seq technology has applications in cardiac hypertrophy [200], HF [201], Atherosclerosis [202], and I/R [203]. However, there are still some technical challenges, such as (1) the isolation of high-quality single cells, which is the most critical step; (2) the performance of clinical biopsy of cardiac samples, which is a difficult problem; (3) the obtaining of sufficient genomic material from single cells, which is critical; and (4) the depth of scRNA-seq transcriptome, which also determines the accuracy of the results. Therefore, single-cell genomics can provide new insights into CVD research, and in-depth research and improvement of this technology can promote new therapies for CVD. MS-based proteomic analyses are broadly grouped into three dominant approaches: bottom-up peptide-centric, top-down protein-centric, and targeted peptide/protein acquisitions. Cardiac proteomics can provide a broader and deeper understanding of the molecular mechanisms underlying cardiovascular diseases and suggest interventions for future treatment. However, nowadays, none of the techniques can be extended to analyze a large number of proteins simultaneously, and none of the techniques have been applied to cardiac problems, which requires further research.

The use of certain HDACi drugs has shown severe cardiac toxicity. This cardiotoxicity includes T-wave flattening, ST-segment depression, and QT interval prolongation, as detected by examining the ECG [204]. QT interval prolongation has been the most severe cardiac event in patients treated with HDACi to date due to its ability to lead to potentially fatal ventricular arrhythmia, known as torsades de pointes [205]. Therefore, it is of great significance to develop drugs that can provide higher efficacy at the site of action, while simultaneously reducing toxicity. Research has found that developing drugs that only act on a single HDAC subtype may provide significant clinical benefits, such as the development of selective inhibitors [206] or HDACi with low hERG affinity [207]. Moreover, among the HDACis currently approved by the FDA, none are specifically indicated for the treatment of CVD. Therefore, it is of great significance to develop drugs that specifically target CVD. The future outlook for HDACis therapy can be concisely encapsulated in three key areas: the enhancement of combination therapy approaches, the innovation of new drug formulations, and the identification of more precise biomarkers [208].

## 5. Summary and Outlook

Starting with cardiovascular diseases such as cardiac hypertrophy, heart failure, atherosclerosis, and myocardial ischemia/reperfusion injury, this paper summarizes the relationship between the roles of HDACs in various protein post-translational modifications, as well as HDACi in post-translational modifications that have been developed to date, aiming to provide new ideas for future research.

It is well known that cardiovascular diseases have a high incidence and morbidity, whereby their prevalence increases significantly with age. Therefore, it is particularly urgent to explore new therapeutic strategies for CVD in view of the rapid aging of the global population. In recent years, protein PTMs were shown to be closely related to the occurrence and development of CVD, which has become a focus of medical research. Most PTMs are reversible and can affect overall health by regulating cell states. PTMs are important in cardiovascular signaling pathways, regulating mitochondrial oxidative stress and inducing cardiomyocyte apoptosis. One of the key mechanisms regulating these modifications is mediated by HDACs. Since the discovery of HDACs and HDACis, researchers have gained a deeper understanding of their roles and potential therapeutic effects. HDACs are a major regulator that controls heart development and promotes stem cell-derived cardiogenesis. However, they also play a key role in mediating cardiac hypertrophy, heart remodeling, and functional recovery after cardiac injury [209]. In recent years, significant progress has been made in understanding the function of HDACs in physiological or pathological heart states as well as their regulatory modes. Although HDACis were initially highlighted as a new class of anticancer drugs, their application scope has gradually expanded to the treatment of various other diseases [210]. Accordingly, HDACs and HDACis critically regulate cardiovascular diseases via the modulation of post-translational modifications. Therefore, the in-depth study of HDACs and their inhibitors is critical. We anticipate that the continued study of HDACs and their inhibitors HDACis in various post-translational modifications will provide important theoretical support and potential targets for the clinical treatment of cardiovascular diseases.

## Figures and Tables

**Table 1:** Four classes of HDAC inhibitors, including their names, chemical structures, and action targets.

Classification	HDAC Inhibitor	Chemical Structure	The Target
Short-chain fatty acid	Sodium Butyrate (NaB)	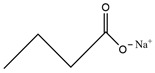	Pan inhibitor
	Valproic acid (VPA)	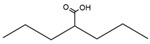	Pan inhibitor
Cyclic peptide	Apicidin	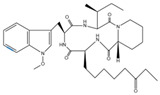	Pan inhibitor
	Romidepsin	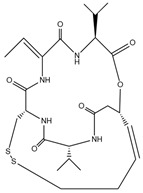	HDAC1/2/4/6
Hydroxamate	Trichostatin A (TSA)	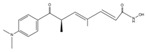	Class I/II
	Vorinostat (SAHA)	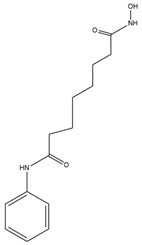	Pan inhibitor
	Belinostat	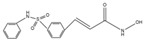	Pan inhibitor
	Panobinostat	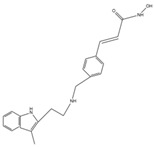	Pan inhibitor
Benzamide	Entinostat	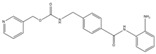	ClassI
	Mocetinostat	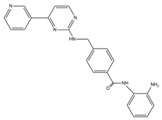	Pan inhibitor

**Table 2:** Effects of HDAC inhibitors in acetylation on cardiovascular disease.

Biological Function	Drug	Target Protein	Modification Site	The Acetylation Level	Reference
Inhibition of cardiac hypertrophy	TSA	HDAC6	H3K9	↓	[173]
	VPA	HDACs		↑	[174]
	Fibroblast Growth Factor 21 (FGF21)	SIRT1		↓	[175]
Atherosclerosis	TSA	HDAC6	H3K9		[176]
Protecting the heart from ischemia/reperfusion injury	Entinostat	HDAC1/2/3		↑	[177]
	SAHA	HDAC1/2/3/6/7/11	H3K14	↑	[178]
	VPA	HDAC1/2	H3K4	↓	[179]
	Tubastatin A (TubA)	HDAC6		↓	[180]
	TSA	HDACs		↑	[181]
	Sodium valproate	HDACs		↑	[182]
	Remifentanil	HDAC3		↑	[183]
	TubA	HDAC6	Prdx1	↑	[150]
Slowing myocardial conduction and increasing susceptibility to refractory arrhythmias	Romidepsin	HDAC1/2/4/6		↓	[184]
	SAHA	HDACs		↑	[185]
Reduction in infarct size in rats with myocardial ischemia/reperfusion injury	Panobinostat	HDAC6		↑	[186]
Reduce myocardial infarction	TSA	HDAC6	H3K9		[187]
Apoptosis and cardiac fibrosis	β-hydroxybutyrate (β-OHB)	HDAC2		↑	[188]

## Data Availability

No new data were created or analyzed in this study.

## References

[1] Chong B., Jayabaskaran J., Jauhari S.M., Chan S.P., Goh R., Kueh M.T.W., Li H., Chin Y.H., Kong G., Anand V.V. (2024). Global burden of cardiovascular diseases: Projections from 2025 to 2050. Eur. J. Prev. Cardiol..

[2] Partridge L., Deelen J., Slagboom P.E. (2018). Facing up to the global challenges of ageing. Nature.

[3] Paneni F., Diaz Cañestro C., Libby P., Lüscher T.F., Camici G.G. (2017). The Aging Cardiovascular System: Understanding It at the Cellular and Clinical Levels. J. Am. Coll. Cardiol..

[4] Malhotra A., Redberg R.F., Meier P. (2017). Saturated fat does not clog the arteries: Coronary heart disease is a chronic inflammatory condition, the risk of which can be effectively reduced from healthy lifestyle interventions. Br. J. Sports Med..

[5] Cheng X., Wang K., Zhao Y., Wang K. (2023). Research progress on post-translational modification of proteins and cardiovascular diseases. Cell Death Discov..

[6] Marquez J., Lee S.R., Kim N., Han J. (2016). Post-Translational Modifications of Cardiac Mitochondrial Proteins in Cardiovascular Disease: Not Lost in Translation. Korean Circ. J..

[7] Jiang G., Li C., Lu M., Lu K., Li H. (2021). Protein lysine crotonylation: Past, present, perspective. Cell Death Dis..

[8] Wang R., Wang G. (2019). Protein Modification and Autophagy Activation. Adv. Exp. Med. Biol..

[9] Yang S., Fan X., Yu W. (2024). Regulatory Mechanism of Protein Crotonylation and Its Relationship with Cancer. Cells.

[10] Grotenbreg G., Ploegh H. (2007). Chemical biology: Dressed-up proteins. Nature.

[11] Geiss-Friedlander R., Melchior F. (2007). Concepts in sumoylation: A decade on. Nat. Rev. Mol. Cell Biol..

[12] Morrison R.S., Kinoshita Y., Johnson M.D., Uo T., Ho J.T., McBee J.K., Conrads T.P., Veenstra T.D. (2002). Proteomic analysis in the neurosciences. Mol. Cell. Proteom..

[13] Fukuda H., Sano N., Muto S., Horikoshi M. (2006). Simple histone acetylation plays a complex role in the regulation of gene expression. Brief. Funct. Genom. Proteomic.

[14] Li X., Foley E.A., Kawashima S.A., Molloy K.R., Li Y., Chait B.T., Kapoor T.M. (2013). Examining post-translational modification-mediated protein-protein interactions using a chemical proteomics approach. Protein Sci..

[15] Chavez J.D., Weisbrod C.R., Zheng C., Eng J.K., Bruce J.E. (2013). Protein interactions, post-translational modifications and topologies in human cells. Mol. Cell. Proteom..

[16] Czuba L.C., Hillgren K.M., Swaan P.W. (2018). Post-translational modifications of transporters. Pharmacol. Ther..

[17] You H., Li S., Chen Y., Lin J., Wang Z., Dennis M., Li C., Yang D. (2023). Global proteome and lysine succinylation analyses provide insights into the secondary metabolism in Salvia miltiorrhiza. J. Proteom..

[18] Choi H., Larsen B., Lin Z.Y., Breitkreutz A., Mellacheruvu D., Fermin D., Qin Z.S., Tyers M., Gingras A.C., Nesvizhskii A.I. (2011). SAINT: Probabilistic scoring of affinity purification-mass spectrometry data. Nat. Methods.

[19] Henikoff S. (2016). Mechanisms of Nucleosome Dynamics In Vivo. Cold Spring Harb. Perspect. Med..

[20] Gregoretti I.V., Lee Y.M., Goodson H.V. (2004). Molecular evolution of the histone deacetylase family: Functional implications of phylogenetic analysis. J. Mol. Biol..

[21] Bradner J.E., West N., Grachan M.L., Greenberg E.F., Haggarty S.J., Warnow T., Mazitschek R. (2010). Chemical phylogenetics of histone deacetylases. Nat. Chem. Biol..

[22] Thiagalingam S., Cheng K.H., Lee H.J., Mineva N., Thiagalingam A., Ponte J.F. (2003). Histone deacetylases: Unique players in shaping the epigenetic histone code. Ann. N. Y. Acad. Sci..

[23] Hsu K.C., Liu C.Y., Lin T.E., Hsieh J.H., Sung T.Y., Tseng H.J., Yang J.M., Huang W.J. (2017). Novel Class IIa-Selective Histone Deacetylase Inhibitors Discovered Using an in Silico Virtual Screening Approach. Sci. Rep..

[24] Demos-Davies K.M., Ferguson B.S., Cavasin M.A., Mahaffey J.H., Williams S.M., Spiltoir J.I., Schuetze K.B., Horn T.R., Chen B., Ferrara C. (2014). HDAC6 contributes to pathological responses of heart and skeletal muscle to chronic angiotensin-II signaling. Am. J. Physiol. Heart Circ. Physiol..

[25] Cheng F., Su L., Yao C., Liu L., Shen J., Liu C., Chen X., Luo Y., Jiang L., Shan J. (2016). SIRT1 promotes epithelial-mesenchymal transition and metastasis in colorectal cancer by regulating Fra-1 expression. Cancer Lett..

[26] Rine J., Strathern J.N., Hicks J.B., Herskowitz I. (1979). A suppressor of mating-type locus mutations in Saccharomyces cerevisiae: Evidence for and identification of cryptic mating-type loci. Genetics.

[27] Tanno M., Sakamoto J., Miura T., Shimamoto K., Horio Y. (2007). Nucleocytoplasmic shuttling of the NAD+-dependent histone deacetylase SIRT1. J. Biol. Chem..

[28] Movahedi Naini S., Sheridan A.M., Force T., Shah J.V., Bonventre J.V. (2015). Group IVA Cytosolic Phospholipase A2 Regulates the G2-to-M Transition by Modulating the Activity of Tumor Suppressor SIRT2. Mol. Cell. Biol..

[29] Nakagawa T., Lomb D.J., Haigis M.C., Guarente L. (2009). SIRT5 Deacetylates carbamoyl phosphate synthetase 1 and regulates the urea cycle. Cell.

[30] Seto E., Yoshida M. (2014). Erasers of histone acetylation: The histone deacetylase enzymes. Cold Spring Harb. Perspect. Biol..

[31] Bagchi R.A., Ferguson B.S., Stratton M.S., Hu T., Cavasin M.A., Sun L., Lin Y.H., Liu D., Londono P., Song K. (2018). HDAC11 suppresses the thermogenic program of adipose tissue via BRD2. JCI Insight.

[32] Marks P.A., Richon V.M., Breslow R., Rifkind R.A. (2001). Histone deacetylase inhibitors as new cancer drugs. Curr. Opin. Oncol..

[33] Glauben R., Siegmund B. (2009). Molecular basis of histone deacetylase inhibitors as new drugs for the treatment of inflammatory diseases and cancer. Methods Mol. Biol..

[34] Zhou C., Zhao D., Wu C., Wu Z., Zhang W., Chen S., Zhao X., Wu S. (2024). Role of histone deacetylase inhibitors in non-neoplastic diseases. Heliyon.

[35] Singh B.N., Zhang G., Hwa Y.L., Li J., Dowdy S.C., Jiang S.W. (2010). Nonhistone protein acetylation as cancer therapy targets. Expert. Rev. Anticancer Ther..

[36] Bush E.W., McKinsey T.A. (2010). Protein acetylation in the cardiorenal axis: The promise of histone deacetylase inhibitors. Circ. Res..

[37] Hsing C.H., Hung S.K., Chen Y.C., Wei T.S., Sun D.P., Wang J.J., Yeh C.H. (2015). Histone Deacetylase Inhibitor Trichostatin A Ameliorated Endotoxin-Induced Neuroinflammation and Cognitive Dysfunction. Mediat. Inflamm..

[38] Trapani D., Esposito A., Criscitiello C., Mazzarella L., Locatelli M., Minchella I., Minucci S., Curigliano G. (2017). Entinostat for the treatment of breast cancer. Expert. Opin. Investig. Drugs.

[39] Ruwizhi N., Aderibigbe B.A. (2020). Cinnamic Acid Derivatives and Their Biological Efficacy. Int. J. Mol. Sci..

[40] Weeks K.L., Gao X., Du X.J., Boey E.J., Matsumoto A., Bernardo B.C., Kiriazis H., Cemerlang N., Tan J.W., Tham Y.K. (2012). Phosphoinositide 3-kinase p110α is a master regulator of exercise-induced cardioprotection and PI3K gene therapy rescues cardiac dysfunction. Circ. Heart Fail..

[41] Nakamura M., Sadoshima J. (2018). Mechanisms of physiological and pathological cardiac hypertrophy. Nat. Rev. Cardiol..

[42] Grimm M., Brown J.H. (2010). Beta-adrenergic receptor signaling in the heart: Role of CaMKII. J. Mol. Cell. Cardiol..

[43] Erickson J.R., Joiner M.L., Guan X., Kutschke W., Yang J., Oddis C.V., Bartlett R.K., Lowe J.S., O’Donnell S.E., Aykin-Burns N. (2008). A dynamic pathway for calcium-independent activation of CaMKII by methionine oxidation. Cell.

[44] Feng N., Anderson M.E. (2017). CaMKII is a nodal signal for multiple programmed cell death pathways in heart. J. Mol. Cell. Cardiol..

[45] Backs J., Backs T., Neef S., Kreusser M.M., Lehmann L.H., Patrick D.M., Grueter C.E., Qi X., Richardson J.A., Hill J.A. (2009). The delta isoform of CaM kinase II is required for pathological cardiac hypertrophy and remodeling after pressure overload. Proc. Natl. Acad. Sci. USA.

[46] He B.J., Joiner M.L., Singh M.V., Luczak E.D., Swaminathan P.D., Koval O.M., Kutschke W., Allamargot C., Yang J., Guan X. (2011). Oxidation of CaMKII determines the cardiotoxic effects of aldosterone. Nat. Med..

[47] Kee H.J., Eom G.H., Joung H., Shin S., Kim J.R., Cho Y.K., Choe N., Sim B.W., Jo D., Jeong M.H. (2008). Activation of histone deacetylase 2 by inducible heat shock protein 70 in cardiac hypertrophy. Circ. Res..

[48] Eom G.H., Cho Y.K., Ko J.H., Shin S., Choe N., Kim Y., Joung H., Kim H.S., Nam K.I., Kee H.J. (2011). Casein kinase-2α1 induces hypertrophic response by phosphorylation of histone deacetylase 2 S394 and its activation in the heart. Circulation.

[49] Grégoire S., Xiao L., Nie J., Zhang X., Xu M., Li J., Wong J., Seto E., Yang X.J. (2007). Histone deacetylase 3 interacts with and deacetylates myocyte enhancer factor 2. Mol. Cell. Biol..

[50] Montgomery R.L., Potthoff M.J., Haberland M., Qi X., Matsuzaki S., Humphries K.M., Richardson J.A., Bassel-Duby R., Olson E.N. (2008). Maintenance of cardiac energy metabolism by histone deacetylase 3 in mice. J. Clin. Investig..

[51] Lin Y.H., Warren C.M., Li J., McKinsey T.A., Russell B. (2016). Myofibril growth during cardiac hypertrophy is regulated through dual phosphorylation and acetylation of the actin capping protein CapZ. Cell Signal.

[52] Zhao T., Kee H.J., Bai L., Kim M.K., Kee S.J., Jeong M.H. (2021). Selective HDAC8 Inhibition Attenuates Isoproterenol-Induced Cardiac Hypertrophy and Fibrosis via p38 MAPK Pathway. Front. Pharmacol..

[53] Karamboulas C., Swedani A., Ward C., Al-Madhoun A.S., Wilton S., Boisvenue S., Ridgeway A.G., Skerjanc I.S. (2006). HDAC activity regulates entry of mesoderm cells into the cardiac muscle lineage. J. Cell Sci..

[54] Davis F.J., Gupta M., Camoretti-Mercado B., Schwartz R.J., Gupta M.P. (2003). Calcium/calmodulin-dependent protein kinase activates serum response factor transcription activity by its dissociation from histone deacetylase, HDAC4. Implications in cardiac muscle gene regulation during hypertrophy. J. Biol. Chem..

[55] Zhang C.L., McKinsey T.A., Chang S., Antos C.L., Hill J.A., Olson E.N. (2002). Class II histone deacetylases act as signal-responsive repressors of cardiac hypertrophy. Cell.

[56] Oliveira R.S., Ferreira J.C., Gomes E.R., Paixão N.A., Rolim N.P., Medeiros A., Guatimosim S., Brum P.C. (2009). Cardiac anti-remodelling effect of aerobic training is associated with a reduction in the calcineurin/NFAT signalling pathway in heart failure mice. J. Physiol..

[57] Miska E.A., Karlsson C., Langley E., Nielsen S.J., Pines J., Kouzarides T. (1999). HDAC4 deacetylase associates with and represses the MEF2 transcription factor. EMBO J..

[58] Madugula K., Mulherkar R., Khan Z.K., Chigbu D.I., Patel D., Harhaj E.W., Jain P. (2019). MEF-2 isoforms’ (A-D) roles in development and tumorigenesis. Oncotarget.

[59] Xu J., Gong N.L., Bodi I., Aronow B.J., Backx P.H., Molkentin J.D. (2006). Myocyte enhancer factors 2A and 2C induce dilated cardiomyopathy in transgenic mice. J. Biol. Chem..

[60] Nakagawa Y., Nishikimi T., Kuwahara K. (2019). Atrial and brain natriuretic peptides: Hormones secreted from the heart. Peptides.

[61] Hohl M., Wagner M., Reil J.C., Müller S.A., Tauchnitz M., Zimmer A.M., Lehmann L.H., Thiel G., Böhm M., Backs J. (2013). HDAC4 controls histone methylation in response to elevated cardiac load. J. Clin. Investig..

[62] McKinsey T.A., Zhang C.L., Olson E.N. (2000). Activation of the myocyte enhancer factor-2 transcription factor by calcium/calmodulin-dependent protein kinase-stimulated binding of 14-3-3 to histone deacetylase 5. Proc. Natl. Acad. Sci. USA.

[63] Chang S., McKinsey T.A., Zhang C.L., Richardson J.A., Hill J.A., Olson E.N. (2004). Histone deacetylases 5 and 9 govern responsiveness of the heart to a subset of stress signals and play redundant roles in heart development. Mol. Cell. Biol..

[64] Vega R.B., Harrison B.C., Meadows E., Roberts C.R., Papst P.J., Olson E.N., McKinsey T.A. (2004). Protein kinases C and D mediate agonist-dependent cardiac hypertrophy through nuclear export of histone deacetylase 5. Mol. Cell. Biol..

[65] McKinsey T.A., Zhang C.L., Olson E.N. (2001). Identification of a signal-responsive nuclear export sequence in class II histone deacetylases. Mol. Cell. Biol..

[66] Harrison B.C., Kim M.S., van Rooij E., Plato C.F., Papst P.J., Vega R.B., McAnally J.A., Richardson J.A., Bassel-Duby R., Olson E.N. (2006). Regulation of cardiac stress signaling by protein kinase d1. Mol. Cell. Biol..

[67] Zhang L., Deng M., Lu A., Chen Y., Chen Y., Wu C., Tan Z., Boini K.M., Yang T., Zhu Q. (2019). Sodium butyrate attenuates angiotensin II-induced cardiac hypertrophy by inhibiting COX2/PGE2 pathway via a HDAC5/HDAC6-dependent mechanism. J. Cell. Mol. Med..

[68] Zhu H., Chan K.T., Huang X., Cerra C., Blake S., Trigos A.S., Anderson D., Creek D.J., De Souza D.P., Wang X. (2022). Cystathionine-β-synthase is essential for AKT-induced senescence and suppresses the development of gastric cancers with PI3K/AKT activation. Elife.

[69] Borradaile N.M., Pickering J.G. (2009). NAD(+), sirtuins, and cardiovascular disease. Curr. Pharm. Des..

[70] Wang S., Wang C., Turdi S., Richmond K.L., Zhang Y., Ren J. (2018). ALDH2 protects against high fat diet-induced obesity cardiomyopathy and defective autophagy: Role of CaM kinase II, histone H3K9 methyltransferase SUV39H, Sirt1, and PGC-1α deacetylation. Int. J. Obes..

[71] Sun W., Liu C., Chen Q., Liu N., Yan Y., Liu B. (2018). SIRT3: A New Regulator of Cardiovascular Diseases. Oxid. Med. Cell. Longev..

[72] Guo L., Yin A., Zhang Q., Zhong T., O’Rourke S.T., Sun C. (2017). Angiotensin-(1-7) attenuates angiotensin II-induced cardiac hypertrophy via a Sirt3-dependent mechanism. Am. J. Physiol. Heart Circ. Physiol..

[73] Yu L., Gong B., Duan W., Fan C., Zhang J., Li Z., Xue X., Xu Y., Meng D., Li B. (2017). Melatonin ameliorates myocardial ischemia/reperfusion injury in type 1 diabetic rats by preserving mitochondrial function: Role of AMPK-PGC-1α-SIRT3 signaling. Sci. Rep..

[74] Chen H.X., Wang X.C., Hou H.T., Wang J., Yang Q., Chen Y.L., Chen H.Z., He G.W. (2023). Lysine crotonylation of SERCA2a correlates to cardiac dysfunction and arrhythmia in Sirt1 cardiac-specific knockout mice. Int. J. Biol. Macromol..

[75] Sadhukhan S., Liu X., Ryu D., Nelson O.D., Stupinski J.A., Li Z., Chen W., Zhang S., Weiss R.S., Locasale J.W. (2016). Metabolomics-assisted proteomics identifies succinylation and SIRT5 as important regulators of cardiac function. Proc. Natl. Acad. Sci. USA.

[76] Pillai V.B., Samant S., Sundaresan N.R., Raghuraman H., Kim G., Bonner M.Y., Arbiser J.L., Walker D.I., Jones D.P., Gius D. (2015). Honokiol blocks and reverses cardiac hypertrophy in mice by activating mitochondrial Sirt3. Nat. Commun..

[77] Reed S.M., Quelle D.E. (2014). p53 Acetylation: Regulation and Consequences. Cancers.

[78] Park S.H., Ozden O., Jiang H., Cha Y.I., Pennington J.D., Aykin-Burns N., Spitz D.R., Gius D., Kim H.S. (2011). Sirt3, mitochondrial ROS, ageing, and carcinogenesis. Int. J. Mol. Sci..

[79] Zhang J., Ren D., Fedorova J., He Z., Li J. (2020). SIRT1/SIRT3 Modulates Redox Homeostasis during Ischemia/Reperfusion in the Aging Heart. Antioxidants.

[80] Luo Y.X., Tang X., An X.Z., Xie X.M., Chen X.F., Zhao X., Hao D.L., Chen H.Z., Liu D.P. (2017). SIRT4 accelerates Ang II-induced pathological cardiac hypertrophy by inhibiting manganese superoxide dismutase activity. Eur. Heart J..

[81] Tang X., Chen X.F., Wang N.Y., Wang X.M., Liang S.T., Zheng W., Lu Y.B., Zhao X., Hao D.L., Zhang Z.Q. (2017). SIRT2 Acts as a Cardioprotective Deacetylase in Pathological Cardiac Hypertrophy. Circulation.

[82] Li Z., Zhang X., Guo Z., Zhong Y., Wang P., Li J., Li Z., Liu P. (2018). SIRT6 Suppresses NFATc4 Expression and Activation in Cardiomyocyte Hypertrophy. Front. Pharmacol..

[83] Sundaresan N.R., Vasudevan P., Zhong L., Kim G., Samant S., Parekh V., Pillai V.B., Ravindra P.V., Gupta M., Jeevanandam V. (2012). The sirtuin SIRT6 blocks IGF-Akt signaling and development of cardiac hypertrophy by targeting c-Jun. Nat. Med..

[84] Shen P., Feng X., Zhang X., Huang X., Liu S., Lu X., Li J., You J., Lu J., Li Z. (2016). SIRT6 suppresses phenylephrine-induced cardiomyocyte hypertrophy though inhibiting p300. J. Pharmacol. Sci..

[85] Vakhrusheva O., Smolka C., Gajawada P., Kostin S., Boettger T., Kubin T., Braun T., Bober E. (2008). Sirt7 increases stress resistance of cardiomyocytes and prevents apoptosis and inflammatory cardiomyopathy in mice. Circ. Res..

[86] Cai Y., Yu S.S., Chen S.R., Pi R.B., Gao S., Li H., Ye J.T., Liu P.Q. (2012). Nmnat2 protects cardiomyocytes from hypertrophy via activation of SIRT6. FEBS Lett..

[87] Abate G., Bastonini E., Braun K.A., Verdone L., Young E.T., Caserta M. (2012). Snf1/AMPK regulates Gcn5 occupancy, H3 acetylation and chromatin remodelling at S. cerevisiae ADY2 promoter. Biochim. Biophys. Acta.

[88] Tanai E., Frantz S. (2015). Pathophysiology of Heart Failure. Compr. Physiol..

[89] Lüscher T.F. (2015). Risk factors for and management of heart failure. Eur. Heart J..

[90] Savarese G., Becher P.M., Lund L.H., Seferovic P., Rosano G.M.C., Coats A.J.S. (2023). Global burden of heart failure: A comprehensive and updated review of epidemiology. Cardiovasc. Res..

[91] Chen Z., Zhong M., Lin Y., Zhang W., Zhu Y., Chen L., Huang Z., Luo K., Lu Z., Huang Z. (2025). METTL7B-induced histone lactylation prevents heart failure by ameliorating cardiac remodelling. J. Mol. Cell. Cardiol..

[92] Wang Y.Y., Gao B., Yang Y., Jia S.B., Ma X.P., Zhang M.H., Wang L.J., Ma A.Q., Zhang Q.N. (2022). Histone deacetylase 3 suppresses the expression of SHP-1 via deacetylation of DNMT1 to promote heart failure. Life Sci..

[93] Travers J.G., Hu T., McKinsey T.A. (2020). The black sheep of class IIa: HDAC7 SIKens the heart. J. Clin. Investig..

[94] Zhang W., Qian S., Tang B., Kang P., Zhang H., Shi C. (2023). Resveratrol inhibits ferroptosis and decelerates heart failure progression via Sirt1/p53 pathway activation. J. Cell. Mol. Med..

[95] Chen L., Li S., Zhu J., You A., Huang X., Yi X., Xue M. (2021). Mangiferin prevents myocardial infarction-induced apoptosis and heart failure in mice by activating the Sirt1/FoxO3a pathway. J. Cell. Mol. Med..

[96] Lin B., Zhao H., Li L., Zhang Z., Jiang N., Yang X., Zhang T., Lian B., Liu Y., Zhang C. (2020). Sirt1 improves heart failure through modulating the NF-κB p65/microRNA-155/BNDF signaling cascade. Aging.

[97] Sun S., Wang C., Weng J. (2021). MicroRNA-138-5p drives the progression of heart failure via inhibiting sirtuin 1 signaling. Mol. Med. Rep..

[98] Lu T.M., Tsai J.Y., Chen Y.C., Huang C.Y., Hsu H.L., Weng C.F., Shih C.C., Hsu C.P. (2014). Downregulation of Sirt1 as aging change in advanced heart failure. J. Biomed. Sci..

[99] Gorski P.A., Jang S.P., Jeong D., Lee A., Lee P., Oh J.G., Chepurko V., Yang D.K., Kwak T.H., Eom S.H. (2019). Role of SIRT1 in Modulating Acetylation of the Sarco-Endoplasmic Reticulum Ca(2+)-ATPase in Heart Failure. Circ. Res..

[100] Wang T., Cao Y., Zheng Q., Tu J., Zhou W., He J., Zhong J., Chen Y., Wang J., Cai R. (2019). SENP1-Sirt3 Signaling Controls Mitochondrial Protein Acetylation and Metabolism. Mol. Cell.

[101] Sundaresan N.R., Bindu S., Pillai V.B., Samant S., Pan Y., Huang J.Y., Gupta M., Nagalingam R.S., Wolfgeher D., Verdin E. (2015). SIRT3 Blocks Aging-Associated Tissue Fibrosis in Mice by Deacetylating and Activating Glycogen Synthase Kinase 3β. Mol. Cell. Biol..

[102] Su H., Cantrell A.C., Chen J.X., Gu W., Zeng H. (2023). SIRT3 Deficiency Enhances Ferroptosis and Promotes Cardiac Fibrosis via p53 Acetylation. Cells.

[103] Castillo E.C., Morales J.A., Chapoy-Villanueva H., Silva-Platas C., Treviño-Saldaña N., Guerrero-Beltrán C.E., Bernal-Ramírez J., Torres-Quintanilla A., García N., Youker K. (2019). Mitochondrial Hyperacetylation in the Failing Hearts of Obese Patients Mediated Partly by a Reduction in SIRT3: The Involvement of the Mitochondrial Permeability Transition Pore. Cell Physiol. Biochem..

[104] Chang X., Zhang T., Wang J., Liu Y., Yan P., Meng Q., Yin Y., Wang S. (2021). SIRT5-Related Desuccinylation Modification Contributes to Quercetin-Induced Protection against Heart Failure and High-Glucose-Prompted Cardiomyocytes Injured through Regulation of Mitochondrial Quality Surveillance. Oxid. Med. Cell. Longev..

[105] Liu S.S., Wu F., Jin Y.M., Chang W.Q., Xu T.M. (2020). HDAC11: A rising star in epigenetics. Biomed. Pharmacother..

[106] Lin Z., Chang J., Li X., Wang J., Wu X., Liu X., Zhu Y., Yu X.Y. (2022). Association of DNA methylation and transcriptome reveals epigenetic etiology of heart failure. Funct. Integr. Genom..

[107] Foks A.C., Bot I. (2017). Preface: Pathology and Pharmacology of Atherosclerosis. Eur. J. Pharmacol..

[108] Famularo G., Trinchieri V., Santini G., De Simone C. (2000). Infections, atherosclerosis, and coronary heart disease. Ann. Ital. Med. Int..

[109] Holmstedt C.A., Turan T.N., Chimowitz M.I. (2013). Atherosclerotic intracranial arterial stenosis: Risk factors, diagnosis, and treatment. Lancet Neurol..

[110] Wolberg A.S., Rosendaal F.R., Weitz J.I., Jaffer I.H., Agnelli G., Baglin T., Mackman N. (2015). Venous thrombosis. Nat. Rev. Dis. Primers.

[111] Hu C., Peng K., Wu Q., Wang Y., Fan X., Zhang D.M., Passerini A.G., Sun C. (2021). HDAC1 and 2 regulate endothelial VCAM-1 expression and atherogenesis by suppressing methylation of the GATA6 promoter. Theranostics.

[112] Desvignes T., Loher P., Eilbeck K., Ma J., Urgese G., Fromm B., Sydes J., Aparicio-Puerta E., Barrera V., Espín R. (2020). Unification of miRNA and isomiR research: The mirGFF3 format and the mirtop API. Bioinformatics.

[113] Fu J., Lu Z.T., Wu G., Yang Z.C., Wu X., Wang D., Nie Z.M., Sheng Q. (2024). Gastrodia elata specific miRNA attenuates neuroinflammation via modulating NF-κB signaling pathway. Int. J. Neurosci..

[114] Lu Y., Wu F. (2018). A new miRNA regulator, miR-672, reduces cardiac hypertrophy by inhibiting JUN expression. Gene.

[115] Tajbakhsh A., Bianconi V., Pirro M., Gheibi Hayat S.M., Johnston T.P., Sahebkar A. (2019). Efferocytosis and Atherosclerosis: Regulation of Phagocyte Function by MicroRNAs. Trends Endocrinol. Metab..

[116] Fasolo F., Di Gregoli K., Maegdefessel L., Johnson J.L. (2019). Non-coding RNAs in cardiovascular cell biology and atherosclerosis. Cardiovasc. Res..

[117] Stojkovic S., Nossent A.Y., Haller P., Jäger B., Vargas K.G., Wojta J., Huber K. (2019). MicroRNAs as Regulators and Biomarkers of Platelet Function and Activity in Coronary Artery Disease. Thromb. Haemost..

[118] Wang H., Sugimoto K., Lu H., Yang W.Y., Liu J.Y., Yang H.Y., Song Y.B., Yan D., Zou T.Y., Shen S. (2021). HDAC1-mediated deacetylation of HIF1α prevents atherosclerosis progression by promoting miR-224-3p-mediated inhibition of FOSL2. Mol. Ther. Nucleic Acids.

[119] Zhao Q., Li S., Li N., Yang X., Ma S., Yang A., Zhang H., Yang S., Mao C., Xu L. (2017). miR-34a Targets HDAC1-Regulated H3K9 Acetylation on Lipid Accumulation Induced by Homocysteine in Foam Cells. J. Cell Biochem..

[120] Mittelstadt M.L., Patel R.C. (2012). AP-1 mediated transcriptional repression of matrix metalloproteinase-9 by recruitment of histone deacetylase 1 in response to interferon β. PLoS ONE.

[121] Kong X., Fang M., Li P., Fang F., Xu Y. (2009). HDAC2 deacetylates class II transactivator and suppresses its activity in macrophages and smooth muscle cells. J. Mol. Cell. Cardiol..

[122] Wang L., Ahn Y.J., Asmis R. (2021). Inhibition of myeloid HDAC2 upregulates glutaredoxin 1 expression, improves protein thiol redox state and protects against high-calorie diet-induced monocyte dysfunction and atherosclerosis. Atherosclerosis.

[123] Zheng B., Han M., Shu Y.N., Li Y.J., Miao S.B., Zhang X.H., Shi H.J., Zhang T., Wen J.K. (2011). HDAC2 phosphorylation-dependent Klf5 deacetylation and RARα acetylation induced by RAR agonist switch the transcription regulatory programs of p21 in VSMCs. Cell Res..

[124] Li X., Chen M., Chen X., He X., Li X., Wei H., Tan Y., Min J., Azam T., Xue M. (2024). TRAP1 drives smooth muscle cell senescence and promotes atherosclerosis via HDAC3-primed histone H4 lysine 12 lactylation. Eur. Heart J..

[125] Chen F., Li J., Zheng T., Chen T., Yuan Z. (2022). KLF7 Alleviates Atherosclerotic Lesions and Inhibits Glucose Metabolic Reprogramming in Macrophages by Regulating HDAC4/miR-148b-3p/NCOR1. Gerontology.

[126] Lee G.H., Park J.S., Jin S.W., Pham T.H., Thai T.N., Kim J.Y., Kim C.Y., Choi J.H., Han E.H., Jeong H.G. (2020). Betulinic Acid Induces eNOS Expression via the AMPK-Dependent KLF2 Signaling Pathway. J. Agric. Food Chem..

[127] Kai H., Wu Q., Yin R., Tang X., Shi H., Wang T., Zhang M., Pan C. (2021). LncRNA NORAD Promotes Vascular Endothelial Cell Injury and Atherosclerosis Through Suppressing VEGF Gene Transcription via Enhancing H3K9 Deacetylation by Recruiting HDAC6. Front. Cell Dev. Biol..

[128] Gorenne I., Kumar S., Gray K., Figg N., Yu H., Mercer J., Bennett M. (2013). Vascular smooth muscle cell sirtuin 1 protects against DNA damage and inhibits atherosclerosis. Circulation.

[129] Yan P., Li Z., Xiong J., Geng Z., Wei W., Zhang Y., Wu G., Zhuang T., Tian X., Liu Z. (2021). LARP7 ameliorates cellular senescence and aging by allosterically enhancing SIRT1 deacetylase activity. Cell Rep..

[130] Dai X., Liu S., Cheng L., Huang T., Guo H., Wang D., Xia M., Ling W., Xiao Y. (2022). Epigenetic Upregulation of H19 and AMPK Inhibition Concurrently Contribute to S-Adenosylhomocysteine Hydrolase Deficiency-Promoted Atherosclerotic Calcification. Circ. Res..

[131] Wang H., He F., Liang B., Jing Y., Zhang P., Liu W., Zhu B., Dou D. (2021). LincRNA-p21 alleviates atherosclerosis progression through regulating the miR-221/SIRT1/Pcsk9 axis. J. Cell. Mol. Med..

[132] Liu B., Zhang B., Guo R., Li S., Xu Y. (2014). Enhancement in efferocytosis of oxidized low-density lipoprotein-induced apoptotic RAW264.7 cells through Sirt1-mediated autophagy. Int. J. Mol. Med..

[133] Mattagajasingh I., Kim C.S., Naqvi A., Yamamori T., Hoffman T.A., Jung S.B., DeRicco J., Kasuno K., Irani K. (2007). SIRT1 promotes endothelium-dependent vascular relaxation by activating endothelial nitric oxide synthase. Proc. Natl. Acad. Sci. USA.

[134] Jung S.B., Kim C.S., Naqvi A., Yamamori T., Mattagajasingh I., Hoffman T.A., Cole M.P., Kumar A., Dericco J.S., Jeon B.H. (2010). Histone deacetylase 3 antagonizes aspirin-stimulated endothelial nitric oxide production by reversing aspirin-induced lysine acetylation of endothelial nitric oxide synthase. Circ. Res..

[135] Schermuly R.T., Ghofrani H.A., Wilkins M.R., Grimminger F. (2011). Mechanisms of disease: Pulmonary arterial hypertension. Nat. Rev. Cardiol..

[136] Jiang W., Wang S., Xiao M., Lin Y., Zhou L., Lei Q., Xiong Y., Guan K.L., Zhao S. (2011). Acetylation regulates gluconeogenesis by promoting PEPCK1 degradation via recruiting the UBR5 ubiquitin ligase. Mol. Cell.

[137] Sun Z., Zhang L., Yin K., Zang G., Qian Y., Mao X., Li L., Jing Q., Wang Z. (2023). SIRT3-and FAK-mediated acetylation-phosphorylation crosstalk of NFATc1 regulates N(ε)-carboxymethyl-lysine-induced vascular calcification in diabetes mellitus. Atherosclerosis.

[138] Velpuri P., Patel P., Yazdani A., Abdi A., Rai V., Agrawal D.K. (2024). Increased Oxidative Stress and Decreased Sirtuin-3 and FOXO3 Expression Following Carotid Artery Intimal Injury in Hyperlipidemic Yucatan Microswine. Cardiol. Cardiovasc. Med..

[139] Yang Z., Huang Y., Zhu L., Yang K., Liang K., Tan J., Yu B. (2021). SIRT6 promotes angiogenesis and hemorrhage of carotid plaque via regulating HIF-1α and reactive oxygen species. Cell Death Dis..

[140] Xu Y., Miao C., Cui J., Bian X. (2021). miR-92a-3p promotes ox-LDL induced-apoptosis in HUVECs via targeting SIRT6 and activating MAPK signaling pathway. Braz. J. Med. Biol. Res..

[141] Huang S., Shao T., Liu H., Wang Q., Li T., Zhao Q. (2022). SIRT6 mediates MRTF-A deacetylation in vascular endothelial cells to antagonize oxLDL-induced ICAM-1 transcription. Cell Death Discov..

[142] Huang J., Dong S., Wu Y., Yi H., Zhang W., Ai X. (2024). Sirtuin 6 Deacetylates Apoptosis-Associated Speck-Like Protein (ASC) to Inhibit Endothelial Cell Pyroptosis in Atherosclerosis. Int. Heart J..

[143] Kawahara T.L., Michishita E., Adler A.S., Damian M., Berber E., Lin M., McCord R.A., Ongaigui K.C., Boxer L.D., Chang H.Y. (2009). SIRT6 links histone H3 lysine 9 deacetylation to NF-kappaB-dependent gene expression and organismal life span. Cell.

[144] Yuan H.F., Zhao M., Zhao L.N., Yun H.L., Yang G., Geng Y., Wang Y.F., Zheng W., Yuan Y., Song T.Q. (2022). PRMT5 confers lipid metabolism reprogramming, tumour growth and metastasis depending on the SIRT7-mediated desuccinylation of PRMT5 K387 in tumours. Acta Pharmacol. Sin..

[145] Luan Y., Liu H., Luan Y., Yang Y., Yang J., Ren K.D. (2022). New Insight in HDACs: Potential Therapeutic Targets for the Treatment of Atherosclerosis. Front. Pharmacol..

[146] Anderson J.L., Morrow D.A. (2017). Acute Myocardial Infarction. N. Engl. J. Med..

[147] Yap J., Cabrera-Fuentes H.A., Irei J., Hausenloy D.J., Boisvert W.A. (2019). Role of Macrophages in Cardioprotection. Int. J. Mol. Sci..

[148] Ibanez B., James S., Agewall S., Antunes M.J., Bucciarelli-Ducci C., Bueno H., Caforio A.L.P., Crea F., Goudevenos J.A., Halvorsen S. (2018). 2017 ESC Guidelines for the management of acute myocardial infarction in patients presenting with ST-segment elevation: The Task Force for the management of acute myocardial infarction in patients presenting with ST-segment elevation of the European Society of Cardiology (ESC). Eur. Heart J..

[149] Du J., Zhang L., Zhuang S., Qin G.J., Zhao T.C. (2015). HDAC4 degradation mediates HDAC inhibition-induced protective effects against hypoxia/reoxygenation injury. J. Cell. Physiol..

[150] Leng Y., Wu Y., Lei S., Zhou B., Qiu Z., Wang K., Xia Z. (2018). Inhibition of HDAC6 Activity Alleviates Myocardial Ischemia/Reperfusion Injury in Diabetic Rats: Potential Role of Peroxiredoxin 1 Acetylation and Redox Regulation. Oxid. Med. Cell. Longev..

[151] Ju J., Li X.M., Zhao X.M., Li F.H., Wang S.C., Wang K., Li R.F., Zhou L.Y., Liang L., Wang Y. (2023). Circular RNA FEACR inhibits ferroptosis and alleviates myocardial ischemia/reperfusion injury by interacting with NAMPT. J. Biomed. Sci..

[152] Eid R.A., Bin-Meferij M.M., El-Kott A.F., Eleawa S.M., Zaki M.S.A., Al-Shraim M., El-Sayed F., Eldeen M.A., Alkhateeb M.A., Alharbi S.A. (2021). Exendin-4 Protects Against Myocardial Ischemia-Reperfusion Injury by Upregulation of SIRT1 and SIRT3 and Activation of AMPK. J. Cardiovasc. Transl. Res..

[153] Xu J.J., Cui J., Lin Q., Chen X.Y., Zhang J., Gao E.H., Wei B., Zhao W. (2021). Protection of the enhanced Nrf2 deacetylation and its downstream transcriptional activity by SIRT1 in myocardial ischemia/reperfusion injury. Int. J. Cardiol..

[154] Tao A., Xu X., Kvietys P., Kao R., Martin C., Rui T. (2018). Experimental diabetes mellitus exacerbates ischemia/reperfusion-induced myocardial injury by promoting mitochondrial fission: Role of down-regulation of myocardial Sirt1 and subsequent Akt/Drp1 interaction. Int. J. Biochem. Cell Biol..

[155] Ning S., Li Z., Ji Z., Fan D., Wang K., Wang Q., Hua L., Zhang J., Meng X., Yuan Y. (2020). MicroRNA-494 suppresses hypoxia/reoxygenation-induced cardiomyocyte apoptosis and autophagy via the PI3K/AKT/mTOR signaling pathway by targeting SIRT1. Mol. Med. Rep..

[156] Ma L., Shi H., Li Y., Gao W., Guo J., Zhu J., Dong Z., Sun A., Zou Y., Ge J. (2021). Hypertrophic preconditioning attenuates myocardial ischemia/reperfusion injury through the deacetylation of isocitrate dehydrogenase 2. Sci. Bull..

[157] Zhang J., He Z., Fedorova J., Logan C., Bates L., Davitt K., Le V., Murphy J., Li M., Wang M. (2021). Alterations in mitochondrial dynamics with age-related Sirtuin1/Sirtuin3 deficiency impair cardiomyocyte contractility. Aging Cell.

[158] Bochaton T., Crola-Da-Silva C., Pillot B., Villedieu C., Ferreras L., Alam M.R., Thibault H., Strina M., Gharib A., Ovize M. (2015). Inhibition of myocardial reperfusion injury by ischemic postconditioning requires sirtuin 3-mediated deacetylation of cyclophilin D. J. Mol. Cell. Cardiol..

[159] Chouchani E.T., Pell V.R., Gaude E., Aksentijević D., Sundier S.Y., Robb E.L., Logan A., Nadtochiy S.M., Ord E.N.J., Smith A.C. (2014). Ischaemic accumulation of succinate controls reperfusion injury through mitochondrial ROS. Nature.

[160] Hu Y., Tian X., Zhao Y., Wang Z., Lin M., Sun R., Wang Y., Wang Z., Li G., Zheng S. (2024). Sirtuin 5 Alleviates Liver Ischemia/Reperfusion Injury by Regulating Mitochondrial Succinylation and Oxidative Stress. Antioxid. Redox Signal.

[161] Xu H., Chen X., Xu X., Shi R., Suo S., Cheng K., Zheng Z., Wang M., Wang L., Zhao Y. (2016). Lysine Acetylation and Succinylation in HeLa Cells and their Essential Roles in Response to UV-induced Stress. Sci. Rep..

[162] Li X., Liu L., Jiang W., Liu M., Wang Y., Ma H., Mu N., Wang H. (2022). SIRT6 Protects Against Myocardial Ischemia-Reperfusion Injury by Attenuating Aging-Related CHMP2B Accumulation. J. Cardiovasc. Transl. Res..

[163] He X., Zeng H., Chen J.X. (2019). Emerging role of SIRT3 in endothelial metabolism, angiogenesis, and cardiovascular disease. J. Cell. Physiol..

[164] He X., Zeng H., Chen S.T., Roman R.J., Aschner J.L., Didion S., Chen J.X. (2017). Endothelial specific SIRT3 deletion impairs glycolysis and angiogenesis and causes diastolic dysfunction. J. Mol. Cell. Cardiol..

[165] Palomer X., Román-Azcona M.S., Pizarro-Delgado J., Planavila A., Villarroya F., Valenzuela-Alcaraz B., Crispi F., Sepúlveda-Martínez Á., Miguel-Escalada I., Ferrer J. (2020). SIRT3-mediated inhibition of FOS through histone H3 deacetylation prevents cardiac fibrosis and inflammation. Signal Transduct. Target. Ther..

[166] Liu G.Z., Xu W., Zang Y.X., Lou Q., Hang P.Z., Gao Q., Shi H., Liu Q.Y., Wang H., Sun X. (2022). Honokiol Inhibits Atrial Metabolic Remodeling in Atrial Fibrillation Through Sirt3 Pathway. Front. Pharmacol..

[167] Qi J., Wang F., Yang P., Wang X., Xu R., Chen J., Yuan Y., Lu Z., Duan J. (2018). Mitochondrial Fission Is Required for Angiotensin II-Induced Cardiomyocyte Apoptosis Mediated by a Sirt1-p53 Signaling Pathway. Front. Pharmacol..

[168] Kong X., Guan J., Li J., Wei J., Wang R. (2017). P66(Shc)-SIRT1 Regulation of Oxidative Stress Protects Against Cardio-cerebral Vascular Disease. Mol. Neurobiol..

[169] Oshikawa J., Kim S.J., Furuta E., Caliceti C., Chen G.F., McKinney R.D., Kuhr F., Levitan I., Fukai T., Ushio-Fukai M. (2012). Novel role of p66Shc in ROS-dependent VEGF signaling and angiogenesis in endothelial cells. Am. J. Physiol. Heart Circ. Physiol..

[170] Paneni F., Mocharla P., Akhmedov A., Costantino S., Osto E., Volpe M., Lüscher T.F., Cosentino F. (2012). Gene silencing of the mitochondrial adaptor p66(Shc) suppresses vascular hyperglycemic memory in diabetes. Circ. Res..

[171] Kumar S., Kim Y.R., Vikram A., Naqvi A., Li Q., Kassan M., Kumar V., Bachschmid M.M., Jacobs J.S., Kumar A. (2017). Sirtuin1-regulated lysine acetylation of p66Shc governs diabetes-induced vascular oxidative stress and endothelial dysfunction. Proc. Natl. Acad. Sci. USA.

[172] Pereira M., Cruz M.T., Fortuna A., Bicker J. (2024). Restoring the epigenome in Alzheimer’s disease: Advancing HDAC inhibitors as therapeutic agents. Drug Discov. Today.

[173] Zhao Z., Lv J., Guo N., Guo Q., Zeng S., Fang Y., Chen W., Wang Z. (2023). Dose-dependent Effects of PRC2 and HDAC Inhibitors on Cardiomyocyte Hypertrophy Induced by Phenylephrine. Curr. Drug Targets.

[174] Kang S.H., Seok Y.M., Song M.J., Lee H.A., Kurz T., Kim I. (2015). Histone deacetylase inhibition attenuates cardiac hypertrophy and fibrosis through acetylation of mineralocorticoid receptor in spontaneously hypertensive rats. Mol. Pharmacol..

[175] Li S., Zhu Z., Xue M., Yi X., Liang J., Niu C., Chen G., Shen Y., Zhang H., Zheng J. (2019). Fibroblast growth factor 21 protects the heart from angiotensin II-induced cardiac hypertrophy and dysfunction via SIRT1. Biochim. Biophys. Acta Mol. Basis Dis..

[176] Gao Q., Wei A., Chen F., Chen X., Ding W., Ding Z., Wu Z., Du R., Cao W. (2020). Enhancing PPARγ by HDAC inhibition reduces foam cell formation and atherosclerosis in ApoE deficient mice. Pharmacol. Res..

[177] Herr D.J., Baarine M., Aune S.E., Li X., Ball L.E., Lemasters J.J., Beeson C.C., Chou J.C., Menick D.R. (2018). HDAC1 localizes to the mitochondria of cardiac myocytes and contributes to early cardiac reperfusion injury. J. Mol. Cell. Cardiol..

[178] Shi X., Yin Y., Guo X., Liu M., Ma F., Tian L., Zheng M., Liu G. (2023). The histone deacetylase inhibitor SAHA exerts a protective effect against myocardial ischemia/reperfusion injury by inhibiting sodium-calcium exchanger. Biochem. Biophys. Res. Commun..

[179] Guerra-Ojeda S., Suarez A., Belmonte B., Marchio P., Genovés P., Arias O.J., Aldasoro M., Vila J.M., Serna E., Mauricio M.D. (2024). Sodium valproate treatment reverses endothelial dysfunction in aorta from rabbits with acute myocardial infarction. Eur. J. Pharmacol..

[180] Williams A.M., He W., Li Y., Bhatti U.F., Nikolian V.C., Chang P., Chang Z., Halaweish I., Liu B., Cheng X. (2018). Histone Deacetylase Inhibition Attenuates Cardiomyocyte Hypoxia-Reoxygenation Injury. Curr. Mol. Med..

[181] Zhao T.C., Cheng G., Zhang L.X., Tseng Y.T., Padbury J.F. (2007). Inhibition of histone deacetylases triggers pharmacologic preconditioning effects against myocardial ischemic injury. Cardiovasc. Res..

[182] Wang D., Fang C., Zong N.C., Liem D.A., Cadeiras M., Scruggs S.B., Yu H., Kim A.K., Yang P., Deng M. (2013). Regulation of acetylation restores proteolytic function of diseased myocardium in mouse and human. Mol. Cell. Proteom..

[183] Chen M., Liu Q., Chen L., Zhang L., Gu E. (2017). Remifentanil postconditioning ameliorates histone H3 acetylation modification in H9c2 cardiomyoblasts after hypoxia/reoxygenation via attenuating endoplasmic reticulum stress. Apoptosis.

[184] Xu Q., Patel D., Zhang X., Veenstra R.D. (2016). Changes in cardiac Nav1.5 expression, function, and acetylation by pan-histone deacetylase inhibitors. Am. J. Physiol. Heart Circ. Physiol..

[185] Colussi C., Berni R., Rosati J., Straino S., Vitale S., Spallotta F., Baruffi S., Bocchi L., Delucchi F., Rossi S. (2010). The histone deacetylase inhibitor suberoylanilide hydroxamic acid reduces cardiac arrhythmias in dystrophic mice. Cardiovasc. Res..

[186] Lin C.F., Hsu K.C., HuangFu W.C., Lin T.E., Huang H.L., Pan S.L. (2020). Investigating the potential effects of selective histone deacetylase 6 inhibitor ACY1215 on infarct size in rats with cardiac ischemia-reperfusion injury. BMC Pharmacol. Toxicol..

[187] Wang Y., Chen P., Wang L., Zhao J., Zhong Z., Wang Y., Xu J. (2018). Inhibition of Histone Deacetylases Prevents Cardiac Remodeling After Myocardial Infarction by Restoring Autophagosome Processing in Cardiac Fibroblasts. Cell Physiol. Biochem..

[188] Xu S., Tao H., Cao W., Cao L., Lin Y., Zhao S.M., Xu W., Cao J., Zhao J.Y. (2021). Ketogenic diets inhibit mitochondrial biogenesis and induce cardiac fibrosis. Signal Transduct. Target. Ther..

[189] David G., Neptune M.A., DePinho R.A. (2002). SUMO-1 modification of histone deacetylase 1 (HDAC1) modulates its biological activities. J. Biol. Chem..

[190] Brandl A., Wagner T., Uhlig K.M., Knauer S.K., Stauber R.H., Melchior F., Schneider G., Heinzel T., Krämer O.H. (2012). Dynamically regulated sumoylation of HDAC2 controls p53 deacetylation and restricts apoptosis following genotoxic stress. J. Mol. Cell. Biol..

[191] Nott A., Watson P.M., Robinson J.D., Crepaldi L., Riccio A. (2008). S-Nitrosylation of histone deacetylase 2 induces chromatin remodelling in neurons. Nature.

[192] Colussi C., Mozzetta C., Gurtner A., Illi B., Rosati J., Straino S., Ragone G., Pescatori M., Zaccagnini G., Antonini A. (2008). HDAC2 blockade by nitric oxide and histone deacetylase inhibitors reveals a common target in Duchenne muscular dystrophy treatment. Proc. Natl. Acad. Sci. USA.

[193] Xu H.D., Wang L.N., Wen P.P., Shi S.P., Qiu J.D. (2018). Site-Specific Systematic Analysis of Lysine Modification Crosstalk. Proteomics.

[194] Hofmann T.G., Möller A., Sirma H., Zentgraf H., Taya Y., Dröge W., Will H., Schmitz M.L. (2002). Regulation of p53 activity by its interaction with homeodomain-interacting protein kinase-2. Nat. Cell Biol..

[195] Sakaguchi K., Herrera J.E., Saito S., Miki T., Bustin M., Vassilev A., Anderson C.W., Appella E. (1998). DNA damage activates p53 through a phosphorylation-acetylation cascade. Genes. Dev..

[196] Appella E., Anderson C.W. (2000). Signaling to p53: Breaking the posttranslational modification code. Pathol. Biol..

[197] Ferguson B.S., Harrison B.C., Jeong M.Y., Reid B.G., Wempe M.F., Wagner F.F., Holson E.B., McKinsey T.A. (2013). Signal-dependent repression of DUSP5 by class I HDACs controls nuclear ERK activity and cardiomyocyte hypertrophy. Proc. Natl. Acad. Sci. USA.

[198] Guttzeit S., Backs J. (2022). Post-translational modifications talk and crosstalk to class IIa histone deacetylases. J. Mol. Cell. Cardiol..

[199] Yu X., Yang X., Cao J. (2023). Application of Single-Cell Genomics in Cardiovascular Research. Cardiol. Ther..

[200] Chaffin M., Papangeli I., Simonson B., Akkad A.D., Hill M.C., Arduini A., Fleming S.J., Melanson M., Hayat S., Kost-Alimova M. (2022). Single-nucleus profiling of human dilated and hypertrophic cardiomyopathy. Nature.

[201] Wang L., Yu P., Zhou B., Song J., Li Z., Zhang M., Guo G., Wang Y., Chen X., Han L. (2020). Single-cell reconstruction of the adult human heart during heart failure and recovery reveals the cellular landscape underlying cardiac function. Nat. Cell Biol..

[202] Kim K., Shim D., Lee J.S., Zaitsev K., Williams J.W., Kim K.W., Jang M.Y., Seok Jang H., Yun T.J., Lee S.H. (2018). Transcriptome Analysis Reveals Nonfoamy Rather Than Foamy Plaque Macrophages Are Proinflammatory in Atherosclerotic Murine Models. Circ. Res..

[203] Gladka M.M., Molenaar B., de Ruiter H., van der Elst S., Tsui H., Versteeg D., Lacraz G.P.A., Huibers M.M.H., van Oudenaarden A., van Rooij E. (2018). Single-Cell Sequencing of the Healthy and Diseased Heart Reveals Cytoskeleton-Associated Protein 4 as a New Modulator of Fibroblasts Activation. Circulation.

[204] Piekarz R.L., Frye R., Turner M., Wright J.J., Allen S.L., Kirschbaum M.H., Zain J., Prince H.M., Leonard J.P., Geskin L.J. (2009). Phase II multi-institutional trial of the histone deacetylase inhibitor romidepsin as monotherapy for patients with cutaneous T-cell lymphoma. J. Clin. Oncol..

[205] Wolbrette D.L. (2004). Drugs that cause Torsades de pointes and increase the risk of sudden cardiac death. Curr. Cardiol. Rep..

[206] Witt O., Deubzer H.E., Milde T., Oehme I. (2009). HDAC family: What are the cancer relevant targets?. Cancer Lett..

[207] Shultz M.D., Cao X., Chen C.H., Cho Y.S., Davis N.R., Eckman J., Fan J., Fekete A., Firestone B., Flynn J. (2011). Optimization of the in vitro cardiac safety of hydroxamate-based histone deacetylase inhibitors. J. Med. Chem..

[208] Shi M.Q., Xu Y., Fu X., Pan D.S., Lu X.P., Xiao Y., Jiang Y.Z. (2024). Advances in targeting histone deacetylase for treatment of solid tumors. J. Hematol. Oncol..

[209] Wang Z., Zhao Y.T., Zhao T.C. (2021). Histone deacetylases in modulating cardiac disease and their clinical translational and therapeutic implications. Exp. Biol. Med..

[210] Bagchi R.A., Weeks K.L. (2019). Histone deacetylases in cardiovascular and metabolic diseases. J. Mol. Cell. Cardiol..

